# Potential Drugs Targeting Early Innate Immune Evasion of SARS-Coronavirus 2 via 2’-*O*-Methylation of Viral RNA

**DOI:** 10.3390/v12050525

**Published:** 2020-05-10

**Authors:** José Antonio Encinar, Javier A. Menendez

**Affiliations:** 1 Institute of Research, Development and Innovation in Biotechnology of Elche (IDiBE) and Molecular and Cell Biology Institute (IBMC), Miguel Hernández University (UMH), 03202 Alicante, Spain; 2 Program Against Cancer Therapeutic Resistance (ProCURE), Metabolism and Cancer Group, Catalan Institute of Oncology, 17005 Girona, Spain; 3 Girona Biomedical Research Institute, 17007 Girona, Spain

**Keywords:** COVID-19, drug repurposing, methylation, methyltransferases, computational screening, molecular docking, molecular dynamics

## Abstract

The severe acute respiratory syndrome coronavirus-2 (SARS-CoV-2) causing the COVID-19 respiratory disease pandemic utilizes unique 2′-O-methyltransferase (2′-O-MTase) capping machinery to camouflage its RNA from innate immune recognition. The nsp16 catalytic subunit of the 2′-O-MTase is unusual in its requirement for a stimulatory subunit (nsp10) to catalyze the ribose 2′-O-methylation of the viral RNA cap. Here we provide a computational basis for drug repositioning or de novo drug development based on three differential traits of the intermolecular interactions of the SARS-CoV-2-specific nsp16/nsp10 heterodimer, namely: (1) the S-adenosyl-l-methionine-binding pocket of nsp16, (2) the unique “activating surface” between nsp16 and nsp10, and (3) the RNA-binding groove of nsp16. We employed ≈9000 U.S. Food and Drug Administration (FDA)-approved investigational and experimental drugs from the DrugBank repository for docking virtual screening. After molecular dynamics calculations of the stability of the binding modes of high-scoring nsp16/nsp10–drug complexes, we considered their pharmacological overlapping with functional modules of the virus–host interactome that is relevant to the viral lifecycle, and to the clinical features of COVID-19. Some of the predicted drugs (e.g., tegobuvir, sonidegib, siramesine, antrafenine, bemcentinib, itacitinib, or phthalocyanine) might be suitable for repurposing to pharmacologically reactivate innate immune restriction and antagonism of SARS-CoV-2 RNAs lacking 2′-O-methylation.

## 1. Introduction

As of 4 May 2020, the pandemic of coronavirus disease 2019 (COVID-19) respiratory disease caused by the pathogenic severe acute respiratory syndrome coronavirus-2 (SARS-CoV-2) has led to more than 3,500,000 confirmed cases and more than 250,000 deaths worldwide [1,2,3,4,5]. Laboratory-based studies using the nucleotide analog remdesivir—a pan-inhibitor of viral RNA-dependent RNA polymerases—and preliminary clinical reports with (hydroxy)chloroquine—an approved, anti-inflammatory drug used to treat malaria, lupus, and rheumatoid arthritis—suggest their potential benefit against SARS-CoV-2 infection and the possible amelioration of viral shedding [6,7,8,9,10,11,12,13]. Accordingly, clinical trials evaluating the survival or time to clinical improvement in severely ill adult patients hospitalized for COVID-19 after adding remdesivir or hydroxychloroquine to standard supportive care, and clinical trials exploring hydroxychloroquine for preventing secondary SARS-CoV-2 transmission following initial contact exposure, are either recruiting or underway. However, no antiviral drugs are yet available with proven efficacy for SARS-CoV-2 treatment or prophylactic strategies to successfully protect individuals at high risk for COVID-19 infection (e.g., close contacts, households, and healthcare workers). 

The current development of novel therapeutics to counteract SARS-CoV-2 infection can be categorized into at least four different strategies, namely: (a) broad-spectrum anti-virals (e.g., remdesivir, ribavirin, cyclophilin, and interferon) [14,15]; (b) drugs targeting the proinflammatory hypercytokinemia (termed “cytokine storm”) driving the transition from first COVID-19 symptoms to acute respiratory distress syndrome (e.g., IL-6 antibody blockers, IL-1 receptor antagonists, and JAK inhibitors) [16,17,18,19,20]; (c) inhibitors of host cell proteases that participate in the priming of the viral Spike (S) glycoprotein [21,22,23,24]; and (d) therapeutics targeting the host–virus interface linking the viral S protein to the angiotensin-converting enzyme 2 (ACE2) receptor in host cells [25,26,27,28,29,30,31,32,33]. In the current pandemic, identifying new targets for already approved drugs (drug repurposing) might shorten the development time and reduce the cost compared with de novo discovery of new compounds targeting one or several of the repertoire of viral proteins (up to 29) [34,35]. The bulk of the drug repurposing efforts seem to be directed toward pharmacologically targeting 3CLpro/nsp5-dependent viral replication [36,37], RdRp/nsp12-driven viral RNA synthesis, and S protein-driven viral cellular entry [22].

**Figure 1:**
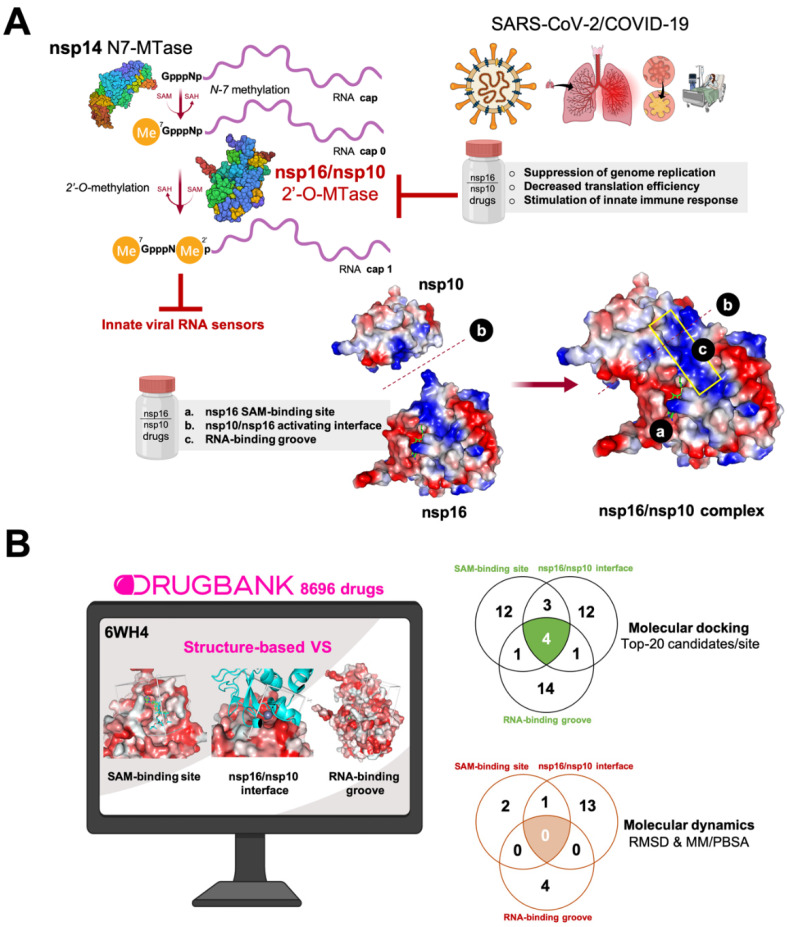
(**A**) Therapeutic relevance of targeting the nsp16/nsp10 2′-O-methyltransferase (2′-O-MTase) protein complex. Severe acute respiratory syndrome coronavirus-2 (SARS-CoV-2) encodes two S-adenosyl-l-methionine (SAM)-dependent methyltransferases (MTases), which sequentially methylate the RNA cap at the guanosine-7 and ribose-2′-O-positions, and are catalyzed by nsp14 N7-MTase and nsp16 2′-O-MTase, respectively. The molecular traits of the coronaviral 2′-O-MTase relative to other eukaryotic MTases make the nsp16/nsp10 protein complex an attractive target for inhibitor design. A unique feature of SARS-CoV-2 is that nsp16 obligatorily requires the non-structural protein nsp10 as a stimulatory factor for exerting its 2′-O-MTase activity by stabilizing the co-substrate SAM-binding pocket and extending the substrate RNA-binging groove. Drugs capable of inhibiting the formation of the nsp16/nsp10 complex (by targeting the nsp16/nsp10 activating interface) and the 2′-O-MTase activity (by targeting the SAM-binding site and the RNA-binding groove) can directly contribute to the suppression of genome replication. The pharmacological reduction of nsp16 2′-O-MTase activity can disrupt the 2′-O-methylation in the RNA cap structure and promote the accumulation of cap-0 structures with lower efficiency of translation, thereby suppressing the synthesis of viral proteins, while the viral RNA lacking a cap-1 structure can be recognized by innate RNA sensors and stimulate an early type I interferon response. (**B**) A computational approach for uncovering SARS-CoV2 2′-O-MTAse-targeting drugs. We performed a structure-based virtual screening (VS) procedure by employing molecular docking of almost 9000 U.S. Food and Drug Administration (FDA)-approved investigational and experimental (discovery-phase) drugs to predict candidates targeting one or many of the three differential traits of the intermolecular interactions of the SARS-CoV-2-specific nsp16/nsp10 heterodimer, namely: a SAM cofactor-binding site slightly different to that of main eukaryotic non-viral MTases; the interface between the stimulatory subunit nsp10 and the catalytic subunit nsp16, which is uniquely different from all other partner-independent 2′-O-MTases; and the (nsp10) allosterically regulated extension and narrowing of the substrate RNA-binding groove. First, we selected the top 20 high-scoring compounds (which therefore have a potentially higher affinity) for each site in a first filtering step; second, based on root mean square deviation (RMSD) values and the molecular mechanics Poisson–Boltzmann surface area (MM/PBSA) parameter, we selected a final list of 20 candidates that were predicted to strongly and stably interact with the abovementioned binding sites of the nsp16/nsp10 complex.

SARS-CoV-2 RNAs are capped at the 5′ end to impede degradation by 5′ exoribonucleases, ensure efficient translation, and evade recognition by the host cell innate immune system [38,39,40,41,42]. Interestingly, the SARS-CoV-2 2′-O-methyltransferase (2′-O-MTase) nsp16 protein is an RNA cap-modifying enzyme that is devoid of enzymatic activity and is activated by nsp10, which interacts with nsp16 and selectively confers upon it 2′-O-MTase activity on N7-methyl guanine RNA caps [43,44,45,46,47]. Thus, the methylation process follows an ordered sequence whereby RNA cap guanine-N7-methyltransferase (N7-MTase, nsp14)-mediated N7-guanine methylation precedes nsp16/nsp10-catalyzed RNA 2′-O-methylation [45,46,47,48,49,50] (Figure 1A). Nsp10 binds nsp16 through a ≈930 Å activation surface in nsp10, a molecular event that promotes nsp16 binding to the capped RNA substrate and the methyl donor S-adenosyl-l-methionine (SAM), stabilizing the SAM-binding pocket and extending the capped RNA-binding groove [45,46,47]. The requirement of nsp10 for nsp16 to execute its 2′-O-MTase activity is a unique feature of SARS-CoV-2 that has not been found in any other virus or host cell. The recently described crystal structure of the nsp16/nsp10 heterodimer has revealed that the nsp16/nsp10 interface and the RNA substrate binding sites may represent better drug targets than the MTase active site for developing highly specific anti-SARS-CoV-2 drugs [45,46]. Crucially, the absence of 2′-O-MTase activity results in a significant attenuation of SARS-CoV infection, which is characterized by decreased viral replication and limited breathing difficulties in animal models [51]. Therefore, pharmacological exploitation of 2′-O-MTase activity might open new treatment and prevention avenues to restore viral RNA recognition and activate intrinsic cell immunity against SARS-CoV-2 (Figure 1A). However, despite the evident therapeutic implications, there have been almost no studies aimed at designing and developing highly specific anti-SARS-CoV-2 drugs impeding the functioning of the RNA cap 2′-O-MTase nsp16/nsp10 protein complex.

Here, we used a virtual screening approach of molecular docking of ≈9000 U.S. Food and Drug Administration (FDA)-approved investigational and experimental (discovery-phase) drugs to identify potential candidates that can be directed to the RNA cap 2′-O-MTase nsp16/nsp10 complex (Figure 1B). To provide a better prediction of the binding modes of the selected drugs to the nsp16/nsp10 protein complex and to explore the stability of the nsp16/nsp10–drug complexes, we performed 100 ns molecular dynamics (MD) simulations and monitored the convergence of the root mean square deviation (RMSD) plots of the nsp16/nsp10 protein complex backbone and the non-hydrogen atoms of the drugs. To further explore the stability of the binding modes of the predicted candidates to the nsp16/nsp10 protein complex, MD simulations were employed for free energy calculations using molecular mechanics Poisson–Boltzmann surface area (MM/PBSA) approximation approaches. Finally, we searched the literature to consider the pharmacological overlapping of the selected, high-scoring nsp16/nsp10-targeting drugs with key functional modules of the virus–host interactome that are relevant for the viral lifecycle, as well as with clinical and laboratory features of COVID-19. 

## 2. Materials and Methods 

### 2.1. Drug Structures

The 3D structures of all the tested compounds belonging to DrugBank Release v.5.1.5. were downloaded from the DrugBank website as SDS files [52] and converted to MOL2 format using Marvin Suite 6.0 tools. Briefly, the script executed from a console (Windows or Linux) was as follows: /ChemAxon/MarvinBeans/bin/molconvert mol 2 /path-SDF-file/3D-structures.sdf -o/path-mol2-files/DBnumber.mol2 -m -g -F. The MOL2 files were then converted to PDBQT files using Open Babel 2.4.1 suite by executing the following script from a console (Windows or Linux): /OpenBabel-2.4.1./obabel/path-mol2-files/DBnumber.mol2 -O /path-pdbqt-files/DBnumber.pdbqt –gen3d -xh -p 7.4.

### 2.2. Viral Proteins Structures

Crystallographic coordinates of the nsp16/nsp10 complex 6W4H with a suitable resolution for docking studies (1.8 Å) were downloaded from the Protein Data Bank (PDB) in the pdb format. The specific edition of protein structures was made using PyMol 2.0 software (PyMOL Molecular Graphics System, v2.3.3 Schrödinger, New York, NY, USA, LLC, at http://www.pymol.org/) without further optimization. The appropriate pH 7.4 protonation state of the nsp17/nsp10 side chains was created using the YASARA structure v19.12.14 software [53].

### 2.3. Molecular Docking Simulation

To perform the docking studies with AutoDockVina (v1.1.2, San Diego, CA, USA), the structures of nsp16 or the nsp16/nsp10 complex were transformed to the PDBQT format, including the atomic charges and atom-type definitions [54,55]. These preparations were performed using the AutoDock/Vina plugin with scripts from the AutoDock Tools package [56]. 

Molecular dockings simulations were carried out in three different regions of the nsp16 protein, namely: the SAM-binding site, the nsp16/nsp10 interface, and the RNA-binding groove. For each region, the grid dimensions were as follows: 23 × 23 × 23 (SAM-binding site), 18 × 25 × 15 (nsp16/nsp10 interface), and 30 × 23 × 30 (RNA groove). AutoDock/Vina was set up on a Linux cluster (see Acknowledgments section), which generates a file for each drug containing the molecular coordinates of up to 20 poses, as well the Gibbs free energy variation (ΔG, kcal/mol) for each pose [57]. 

### 2.4. Molecular Dynamics Simulations 

YASARA dynamics v19.9.17 (Vienna, Austria) was employed to carry out all the MD simulations with AMBER14 as a force field. The simulation cell was allowed to include 20 Å surrounding the protein that was filled with water at a density of 0.997 g/mL. Initial energy minimization was carried out under relaxed constraints using steepest descent minimization. Simulations were performed in water under constant pressure and constant temperature (25 °C) conditions. To mimic a physiological environment, counter ions were added to neutralize the system (Na^+^ or Cl^−^ were added as a replacement for water to give a final NaCl concentration of 0.9% and the pH was maintained at 7.4). Hydrogen atoms were added to the protein structure at the appropriate ionizable groups according to both the calculated pK_a_ and the simulation pH (i.e., a hydrogen atom was added if the computed pK_a_ was higher than the pH). The pK_a_ was computed for each residue according to the Ewald method [58]. All simulation steps were run using a pre-installed macro (md_run.mcr) within the YASARA suite. Data were collected every 100 ps. The molecular mechanics Poisson–Boltzmann surface area (MM/PBSA) calculations were used to determine the alchemical binding free energy of drug candidates against nsp16 using the YASARA macro md_analyzebindenergy.mcr, as previously described [59,60,61].

### 2.5. Evaluation of nsp16-Drug Interactions

The best poses of the predicted drug candidates with the best affinity scores were merged with the optimized protein structure in the pdb format using PyMol 2.0 software (New York, NY, USA). Contact residues of the merged structures were analyzed using the protein–ligand interaction profiler (PLIP) algorithm. 

## 3. Results

**Figure 2:**
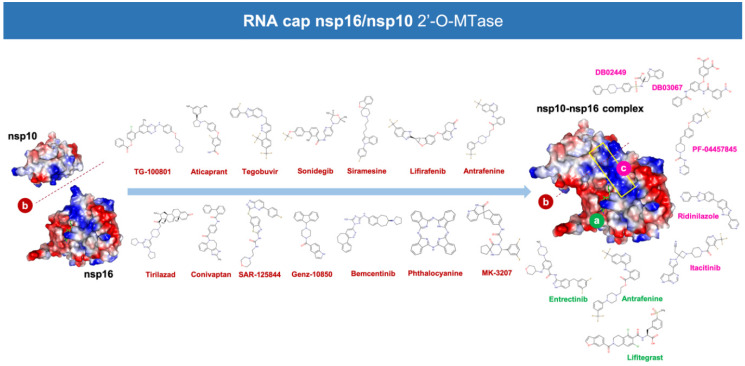
Predicted potential inhibitors of the nsp16/nsp10 2′-O-MTAse complex. Structures of the potential drugs were predicted to target: (**a**) the (nsp10-stabilized) S-adenosyl-l-methionine (SAM)-binding pocket of nsp16 (green code), (**b**) the unique “activating surface” between nsp16 and nsp10 (brown code), and (**c**) the (nsp10-extended) RNA-binding groove of nsp16 (pink code).

We performed structure-based virtual screening involving molecular docking and MD strategies to select putative candidates among 8696 drugs in the DrugBank database. The target employed was 6W4H, the 1.80 Å resolution crystal structure of the SARS-CoV-2 nsp16/nsp10 complex that was recently released (2020-03-18) by the Center for Structural Genomics of Infectious Diseases (www.csgid.org). The database was docked into three different binding sites of the nsp16/nsp10 complex, namely: the SAM-binding site, the nsp16/nsp10 interface, and the RNA-binding groove (Figure 2). Once the conformational search algorithm probed the energy landscape of each drug in each binding site [54,55] (http://shaker.umh.es/Publications/PDF/nsp16/DrugBank-nsp16-docking.zip), we selected the top 20 high-scoring compounds (which therefore have potentially higher affinity) for each site in a first filtering step. The selected compounds exhibited binding energy values below −10 kcal/mol in the SAM-binding site, below −8 kcal/mol in the nsp16/nsp10 interface, and below −9 kcal/mol in the RNA binding groove (Table S1), thus representing K_D_ (K_D_ = exp^ΔG/RT^) values in the nanomolar to low-micromolar range. The compounds were then grouped based on interactions (i.e., predicted bioactivity) with the same (or different) binding sites (Figure 1B). 

Based on the results of the molecular docking calculations, twelve drugs were predicted to occupy the SAM binding site with a high affinity (Table S2), twelve were predicted to occupy the nsp16/nsp10 interface with a high affinity (Table S3), fourteen were predicted to occupy the RNA-binding groove with a high affinity (Table S4), three were predicted to occupy both the nsp16/nsp10 interface and the SAM binding site with a high affinity (Table S5), one was predicted to dually target the SAM-binding site and the RNA-binding groove (Table S6), one was predicted to dually target the interface and the RNA-binding groove (Table S7), and four were predicted to occupy all the tested cavities of the nsp16/nsp10 protein complex with a high affinity (Table S8). 

Because the flexibility of the target-binding site during the molecular recognition process is an essential but frequently overlooked aspect to be considered in molecular docking, we approached such flexibility issues using MD techniques. Accordingly, we performed MD simulations for 100 ns for each of the high-scoring nsp16/nsp10–drug complexes to confirm the kinetic stability of the poses and validate the binding poses obtained by docking. The nsp16/nsp10 protein backbone RMSD plots of the drugs’ heavy atoms, measured after superimposing nsp16/nsp16 on its reference structure during the MD simulation, were prepared in parallel. A second filter stage considered the stable behaviors of the drug–protein complexes when RMSD values did not exceed 10–15 Å and the starting and ending poses of the drug were similar throughout the MD simulation. Those drugs that did not meet such requirements were discarded as potential candidates (Table S9). To further verify the stability of the nsp16/nsp10–drug complexes, we evaluated the MM/PBSA parameters, which estimate the free energy of the binding of small ligands to biological macromolecules, using MD simulations of the receptor–ligand complex; the results showed good correlations with the experimentally obtained values despite excluding the conformational entropy or the number and free energy of water molecules in the binding site [62,63]. In this third filter, a well-recognized scoring function that commonly serves as a powerful tool in the correct ranking of drug candidates, which is the fact that more positive energies indicate the occurrence of stronger binders. We finally combined the second and third filtering criteria to generate a definitive list of candidates that were classified as either “strong” (>25 kcal/mol) or “weak” (<25 kcal/mol) (Table S10). 

Twenty different compounds were cataloged as strong candidates, with three of them were predicted to stably target the SAM-binding site, fourteen of them were predicted to stably target the nsp16/nsp10 interface, and four of them were predicted to stably target the RNA binding groove (Figure 2).

**Figure 3:**
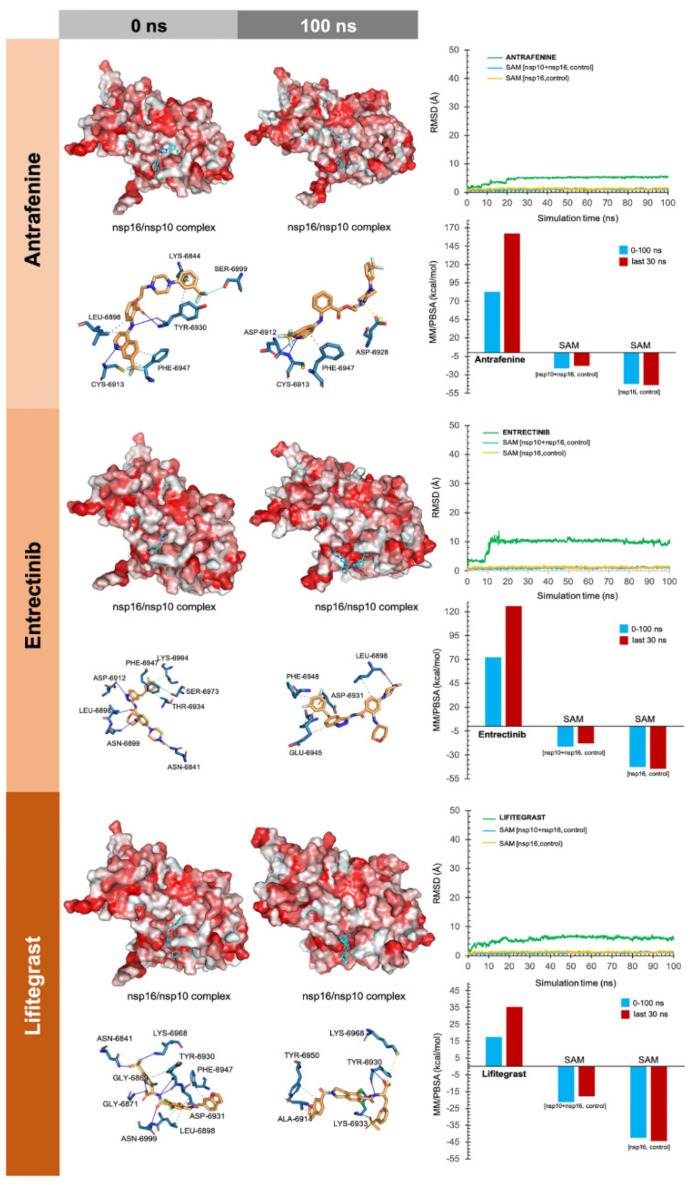
Incorporation models of the predicted SAM-binding-site-targeting drugs in SARS-CoV-2 2′-O-MTAse. The best poses of the predicted candidates coupled to the SAM-binding site of the nsp16/nsp10 complex before (0 ns) and after (100 ns) the molecular dynamics (MD) simulation are shown. The protein is represented as a function of the hydrophobicity of its surface amino acids and the Na^+^ and Cl^−^ ions have been eliminated to facilitate visualization. Each inset shows the detailed interactions of each drug candidate docked to the SAM-binding site of nsp16, indicating the participating amino acids involved in the interaction and the type of interaction (hydrogen bonds, hydrophilic interactions, salt bridges, Π-stacking, etc). The root mean square deviation (RMSD, Å) of each drug’s heavy atoms over the simulation time, measured after superposing the protein on its reference structure, is shown. In the context of the MD presented here, the RMSD incorporates traces of SAM in two independent control simulations (i.e., nsp16/nsp10 complex and nsp16 alone).

**Figure 4:**
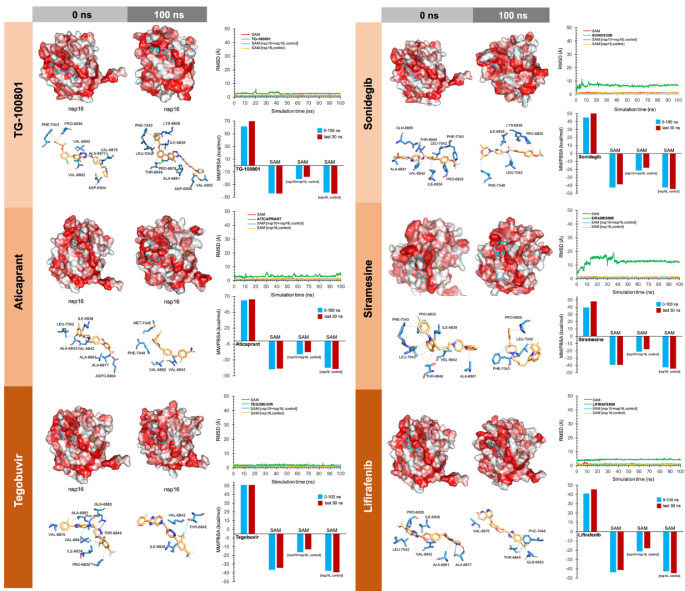
Incorporation models of the predicted nsp16-activating-surface-targeting drugs in SARS-CoV-2 2′-O-MTAse. The best poses of the predicted candidates coupled to the nsp16 activating surface before (0 ns) and after (100 ns) the molecular dynamics (MD) simulation are shown. The protein has been represented as a function of the hydrophobicity of its surface amino acids and the Na^+^ and Cl^−^ ions have been eliminated to facilitate visualization. Each inset shows the detailed interactions of each drug candidate docked to the nsp16 activating surface, indicating the participating amino acids involved in the interaction and the type of interaction (hydrogen bonds, hydrophilic interactions, salt bridges, Π-stacking, etc). The root mean square deviation (RMSD, Å) of each drug’s heavy atoms over the simulation time, measured after superposing the protein on its reference structure, is shown. In the context of the MD presented here, the RMSD incorporates traces of SAM alone and in two additional control simulations (i.e., nsp16/nsp10 complex and nsp16 alone).

**Figure 5:**
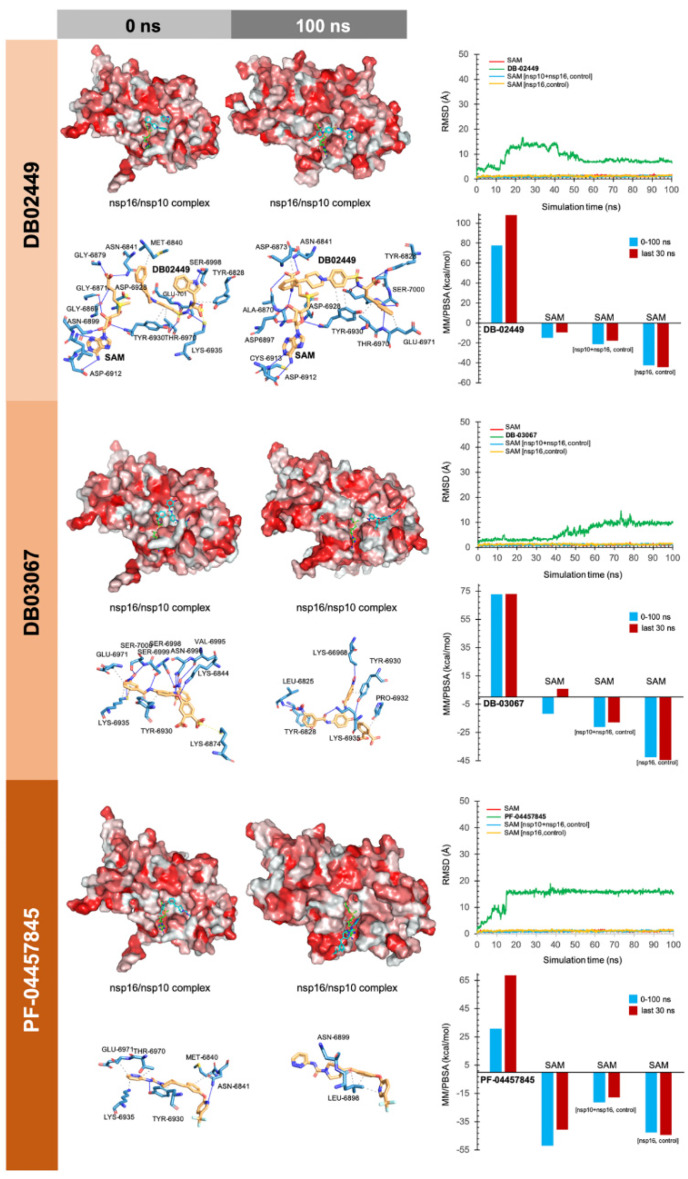
Incorporation models of predicted RNA-binding-groove-targeting drugs in SARS-CoV-2 2′-O-MTAse. The best poses of predicted candidates coupled to the RNA activating surface of nsp16 before (0 ns) and after (100 ns) the molecular dynamics (MD) simulation are shown. The protein has been represented as a function of the hydrophobicity of its surface amino acids and the Na^+^ and Cl^-^ ions have been eliminated to facilitate visualization. Each inset shows the detailed interactions of each drug candidate docked to the RNA-binding groove of nsp16, indicating the participating amino acids involved in the interaction and the type of interaction (hydrogen bonds, hydrophilic interactions, salt bridges, Π-stacking, etc). The root mean square deviation (RMSD, Å) of each drug’s heavy atoms over the simulation time, measured after superposing the protein on its reference structure, is shown. In the context of the MD presented here, the RMSD incorporates traces of SAM alone and in two additional control simulations (i.e., nsp16/nsp10 complex and nsp16 alone).

To facilitate the understanding of the predicted binding modes of the candidates selected in Table S2, we have illustrated the detailed interactions of nsp16 (or the nsp16/nsp10 complex) with the SAM-binding-site-targeting drugs antrafenine, entrectinib, and lifitegrast (Figure 3), with the nsp16/nsp10 interface-targeting drugs TG-100801, LY-2456302, tegobuvir, sonidegib, siramesine, and lifirafenib (Figure 4), and with the RNA binding groove-targeting drugs DB02449, DB03067, and PF-04457845 (Figure 5). Such depictions include the MS simulations at 0 and 100 ns, the time evolution of RMSD relative to the initial structure of nsp16/nsp10 in the absence and presence of drug candidates, MM/PBSA binding energy analyses calculated from the trajectory of the 100 ns (or last 30 ns) MD simulation, and identification of amino acid residues participating in the drug-binding pocket. 

As internal controls, RMSD-based evaluation of the protein’s thermodynamic stability during the MD simulation included not only SAM but also the nsp16-SAM and/or nsp16/nsp10–SAM complex; given that SAM is a methyl donor for the methylation reaction that operates as an nsp16 coenzyme, it is not expected to stabilize the protein structure. The calculated MM/PBSA values of the nsp16–SAM complex in the absence of nsp10 notably increased in its presence, thereby in silico confirming that the partnering of nsp10 stabilizes the interaction of SAM with the catalytic nsp16 [45,46]. Drug candidates such as antrafenine, entrectinib, and lifitegrast are predicted to compete with SAM for the SAM-binding pocket of nsp16 (Figure 3), thereby impeding its methyltransferase activity. Drug candidates such as TG-100801, LY-2456302, tegobuvir, sonidegib, siramesine, and lifirafenib are predicted to bind tightly (mostly via hydrophobic interactions) to the activating interface of nsp16 (Figure 4), thereby impeding the obligatory formation of the active nsp16/nsp10 complex. Drug candidates such as DB02449, DB03067, and PF-04457845 are predicted to stably occupy the RNA groove, mostly via hydrophobic interactions and hydrogen bonds (Figure 5), thereby preventing a proper positioning of the viral RNA that enables its capping. 

## 4. Discussion

The SARS-CoV-2 causing the current pandemic of COVID-19 respiratory disease tends to have a longer incubation period (2–11 days) than the influenza virus (1–4 days). This long prodromal phase, which significantly increases the pre- and oligosymptomatic virus transmission capability of infected individuals, is largely due to its ability to efficiently evade immune detection and to dampen human immune responses. This adaptation of SARS-CoV-2 to escape recognition and activation of the host immune response involves the RNA cap modifying enzyme 2′-O-MTase, which provides the viral mRNA the ability to camouflage itself from the host cell [64]. Accordingly, human coronavirus mutants lacking 2′-O-MTase are exquisitely sensitive to a higher expression of induced type-I interferons [40,41,42]. However, despite the strong rationale for the development of anti-SARS-CoV-2 interventions, such as live attenuated vaccines (e.g., employing 2′-O-methylation RNA mutants) and small-molecule inhibitors, very few attempts have been made to reactivate the innate immune restriction and antagonism of SARS-CoV-2 RNAs lacking 2′-O-methylation via the pharmacological blockade of the coronaviral 2-O′-MTase.

Here, we provide a first-in-class computational basis for drug repositioning or de novo drug development based on three differential traits of the intermolecular interactions of the RNA cap 2′-O-MTase nsp16/nsp10 protein complex, namely: the (nsp10-stabilized) SAM-binding pocket of nsp16, the (nsp10-extended) RNA-binding groove of nsp16, and the unique nsp16/nsp10 interaction interface that is obligatorily required by nsp16 to execute its 2′-O-MTase activity [44,45,46,47]. We provide a careful description and a ranked list of compounds that can be tested experimentally. Some of the identified small molecules might be repurposed or be considered as potential lead molecules for further optimization and drug development against SARS-CoV-2. Of the initially selected 47 docking candidates, only 20 were considered further as strong candidates upon review of the MD/RMSD stability criteria (Figure 1B). We acknowledge that such conventional MD simulations of nanosecond time scales, which will require enhanced sampling methods to provide a complete picture of the key residues used for drug candidates for effective nsp16/nsp10 inhibition, should be viewed as a preliminary approach to identify moieties critical for the retention of key nsp16/nsp10–drug interactions. Nevertheless, they may be useful when analyzing the predicted candidates as lead molecules for optimization and drug development against the nsp16/nsp10 complex. We also acknowledge that some of the predicted drugs, especially those for the treatment of tumors, are expected to produce serious side-effects, and in some cases, our data inferred that high concentrations are required to inhibit nsp16/nsp10 effectively. Accordingly, these drugs may not be considered safe therapies for a population of infected/sick people. Furthermore, while computational approaches offer novel testable hypotheses for systematic drug repositioning, it can be argued that single virus proteins will carry a high risk of drug resistance given the rapid evolution of virus genomes. 

**Figure 6:**
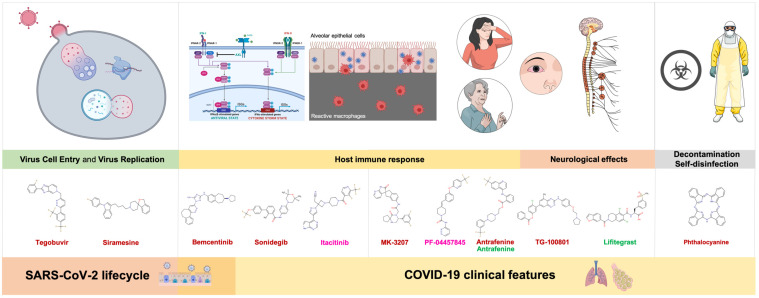
Potential repurposed drug candidates against SARS-CoV2 2′-O-MTAse: a multi-layer network framework. Upon the selection of strong candidates based on the stability of high-scoring nsp16/nsp10–drug complexes (Table S2; Figure 3), we re-evaluated their pharmacological overlapping with functional modules of the virus–host interactome relevant for the viral lifecycle, as well as with clinical and laboratory features of COVID-19. The proposed multi-layer network framework might help to select the narrowest list of candidates that can be rapidly tested experimentally before evaluating their in vivo efficiency and side-effects.

Because viruses require host cellular factors for successful replication, the systematic identification of the so-called virus–host protein–protein interactome [34,65] would be expected to provide key insights into effective molecular targets for developing broadly acting effective treatments against SARS-CoV-2 and other upcoming coronavirus strains. A recent landmark systematic mapping of the interaction landscape between SARS-CoV-2 proteins and human proteins provided the interaction map of nsp10 [66]. Unfortunately, the authors were unable to generate data for the nsp16 protein. Despite this, and with the intention of providing a broader mechanistic context to allow investigators to experimentally or clinically pursue drugs directly, we decided to carefully re-evaluate the selected anti-nsp10/nsp16 candidates in terms of not only their pharmacological overlapping with key functional modules of the SARS-CoV-2-human interactome relevant for the viral lifecycle but also with clinical and laboratory features of COVID-19 (Figure 6). Based on such a comprehensive evaluation, we prioritized several candidates, as described below. 

### 4.1. Virus Cell Entry and Replication

#### 4.1.1. Tegobuvir

Tegobuvir (GS-9190), a novel inhibitor of the hepatitis C virus (HCV) RNA replication in vitro, has shown potential antiviral activity in patients chronically infected with genotype 1 HCV [67,68,69,70]. A recent docking-based repurposing approach predicted the ability of tegobuvir to target the SARS-CoV-2 core RNA-synthesis machinery by occupying the interface between the nsp12 polymerase and its essential co-factor nsp7 [71]. Our present in silico approach predicted the ability of tegobuvir to occupy the activating interface of the nsp16 2′-O-MTase and its essential co-factor nsp10. It is noteworthy that ribavirin, a prodrug that is metabolized to nucleoside analogs to block viral RNA synthesis and viral mRNA capping proposed as an anti-viral therapy against SARS-CoV-2 based on in vitro data [72,73], was previously demonstrated to exhibit limited efficacy against coronaviruses in vivo [74]. This is because the coronaviral nsp14 protein, a bifunctional enzyme carrying RNA cap guanine N7-MTase and 3′-5′ exoribonuclease activities, associates with the low-fidelity nsp12 viral RNA polymerase, thereby generating a robust RNA synthesis and proofreading pathway in which the antiviral ribavirin 5′-monophosphate is significantly incorporated but also readily excised from RNA. Tegobuvir might be worthy of further investigation as a non-nucleoside inhibitor of RNA synthesis and 2′-O-methylation of viral mRNA to attenuate SARS-CoV-2 replication. 

#### 4.1.2. Siramesine

The gold standard sigma-2 receptor agonist siramesine (formerly known as Lu-28-179) was among the high-scoring candidates predicted to stably occupy the activating surface occurring between nsp10 and nsp16. Siramesine is a direct lysosomal toxin that disrupts the lysosomal pH gradient, which is likely done by targeting vacuolar V-ATPase in the endosomal/lysosome membrane, thereby triggering lysosomal dysfunction/destabilization and the accumulation of autophagosomes to counteract the lysosomal cell death pathway [75,76,77,78,79]. Coronaviruses, such as SARS-CoV-2, interact differentially with the autophagy machinery to utilize components for virus replication while attenuating autophagy per se to promote viral production [80]. Compounds such as siramesine, which can reinstate cytoprotective autophagy, might reduce viral production. Moreover, siramesine-related drugs, such as the sigma-1 receptor agonist chloroquine [81,82] and the endo/lysosomal V-ATPase inhibitor bafilomycin A1, may also promote viral trapping with perinuclear vacuoles and interference with terminal glycosylation of the SARS-CoV-2 cellular receptor ACE2 [83,84]. Siramesine belongs to the group of lysosomotropic agents with potential therapeutic efficacy against COVID-19 by impacting cellular trafficking through the endosome/lysosome endocytic pathways and by promoting the accumulation of autophagosomes, which associate with a rapid and sustained inhibition of mTOR, a negative regulator of autophagy [76,77]. 

It should be noted that other SARS-CoV-2 non-structural proteins, such as the virus replication component nsp6, have been shown to interact with sigma receptors [66], which are also targeted (albeit with lower affinity) by therapeutic candidates for COVID-19 treatment and the prevention of secondary SARS-CoV-2 transmission, such as (hydroxy)chloroquine. Future work should evaluate the apparently central role of the sigma-receptor-regulated endoplasmic reticulum stress/unfolded protein response pathway [85,86,87], not only as a major aspect of the coronavirus-host interactome but also in the mechanistic context of sigma-receptor-targeting drugs with therapeutic potential against SARS-CoV-2. 

Siramesine was originally designed to treat anxiety and depression [88,89]. Although clinical trials failed to show satisfactory efficacy in the treatment of psychiatric disorders, the drug was well-tolerated and non-toxic in humans. The lack of side-effects; the strong and immediate effects of siramesine on lysosome acidification, trafficking, and autophagy activation; and its predicted capacity to target the nsp16/nsp10 interface might open new avenues for the therapeutic consideration of this lysosomotropic agent and/or closely related sigma-2-receptor-targeting drugs [66,90,91,92].

### 4.2. Host Immune Response

#### 4.2.1. Bemcentinib

Bemcentinib (BGB324/R-428), a first-in-class highly selective oral AXL tyrosine kinase inhibitor in phase II development, was also predicted to stably occupy the nsp16/nsp10 activating interface. Although mainly recognized for its ability to block epithelial–mesenchymal transition phenomena in various cancer types [93,94,95], bemcentinib is now known for its anti-inflammatory and anti-fibrotic roles in non-cancer diseases [96,97], which are two phenotypic aspects that might be relevant in the therapeutic management of COVID-19. Moreover, AXL is a family member of the TAM receptor tyrosine kinases that operates both as a pleiotropic inhibitor of the innate immune response and a docking site during the cellular entry of some viruses [98,99,100]. As AXL receptor signaling participates in the downmodulation of interferon-related host immune responses to promote viral infection, inhibiting AXL function might be a new therapeutic avenue toward reducing the transmission of some of the most troublesome emerging enveloped viruses, including dengue, West Nile, Ebola, lentivirus, and most recently, Zika [99,100]. If experimentally confirmed as a blocker of the nsp16/nsp10-driven viral innate immunoevasion via suppression of viral RNA 2′-O-methylation, bemcentinib might also stimulate an advantageous increase in the early expression of interferon-stimulated genes that are capable of promoting a greater attenuation of SARS-CoV-2 replication. Bemcentinib was recently shown to block lysosomal acidification and promote the accumulation of autophagosomes in cancer cells independently of its inhibitory effects on AXL [101,102], thereby suggesting an additional overlapping mechanism with the above-mentioned lysosomotropic agents.

#### 4.2.2. Sonidegib

Sonidegib (Odomzo^®^), an FDA-approved oral, selective smoothened (SMO) inhibitor used to treat basal cell carcinomas via regulation of the hedgehog (Hh) signaling pathway [103,104,105] was among the best drug candidates predicted to impede the formation of the obligatory nsp16/nsp10 complex by stably occupying the nsp16 activating surface. The Hh signaling is targeted by numerous viral pathogens to control the local infected environment [106]. Due to the key roles of Hh signaling in tissue repair processes and immunity (e.g., immune cell proliferation and activation), the ability of viral pathogens (e.g., influenza, Epstein-Barr virus, Hepatitis B and C virus, human immunodeficiency virus) that cause damage to the host tissue can exacerbate such Hh-driven responses (e.g., activation of STAT3 and upregulation of IL-6 expression) to drive a detrimental outcome, including fibrosis and an imbalanced immune response [106]. Accordingly, the therapeutic use of molecules inhibiting Hh signaling, such as sonidegib, might be expanded for use as potent, broad-spectrum inhibitors of pathogenic infections by targeting highly-conserved host targets also shared by SARS-CoV-2/COVID-19. 

#### 4.2.3. Itacitinib

Itacitinib is a JAK1-selective inhibitor of the JAK/STAT pathway with >20-fold selectivity over JAK2 and a >100-fold selectivity over JAK3 and TYK2 [107,108]. Cell and animal models and phase II studies have shown potent, pre- and clinical efficacy of itacitinib against psoriasis and rheumatoid arthritis in a JAK2-independent manner [109,110,111,112,113]. As with other JAK inhibitors, such as baricitinib or tofacitinib, itacitinib is being studied for the prevention of the so-called cytokine release syndrome (CRS) or cytokine storm syndrome (CSS) [114], an acute systemic inflammatory syndrome characterized by fever and multiple organ dysfunction [115]. It is now known that a subgroup of patients with severe COVID-19 have had a CSS that occurred 7–10 days after the disease onset, coinciding with the peak of respiratory distress [14,15,116,117]; immune dysregulation, rather than the level of peak viremia, is responsible for the SARS-CoV-2 infection driving a too-little-and-too-late type-I-interferon response accompanied by aberrant proinflammatory cytokine secretion by alveolar macrophages and subsequent CD4^+^ and CD8^+^ T-cell dysfunction. In this life-threatening clinical scenario of hyperinflammation with dysregulated and profuse immune responses causing lung damage, the use of antivirals as single-agents may be insufficient to stop the cytokine storm, pulmonary destruction, and respiratory distress in patients. Accordingly, pharmacological suppression of hypercytokinemia using a selective cytokine blockade and JAK inhibition could improve mortality. Targeted immunomodulation with the dual JAK1/JAK2 inhibitor baricitinib is currently being evaluated in clinical trials aimed at reducing the SARS-CoV-2-driven CCS, ameliorating pulmonary inflammation, and improving mortality [118,119]. Because different JAK inhibitors modulate distinct cytokine pathways to varying degrees but fail to potently or continuously inhibit an individual cytokine pathway [111,113], further clinical studies are warranted to explore whether nsp16/nsp10 inhibition using a JAK inhibitor, such as itacitinib, might provide a means to first re-activate an innate immune response (via nsp16/nsp10 targeting) and later provide prophylactic prevention against the CSS.

### 4.3. Neurological Effects

#### 4.3.1. MK-3207, PF-04457845, and Antrafenine

The small-molecule inhibitor of the CGRP receptor belonging to the “gepant” class of compounds for acute migraine therapy MK-3207 [120,121,122,123,124,125] was among the high-scoring candidates predicted to target the nsp16/nsp10 2′-O-MTase complex. A significant number of patients with COVID-19 show neurologic manifestations, including not only stroke and encephalopathy but also headache, seizures, nausea, olfactory disorders, and vomiting [126,127,128,129], which are also common symptoms of acute migraines. Recent reports of profound myocarditis and fatal arrhythmias have suggested the potential influence of SARS-CoV-2 on the cardiovascular system [130,131,132]. The neuroinvasive potential of SARS-CoV-2, which has been shown to invade the central nervous system and to infect the brainstem [133,134], may play an unexpected role in the respiratory distress and cardiovascular impact of COVID-19. Coronaviruses, such as SARS-CoV-2, can travel retrograde along neuronal synapses from the mechanoreceptors and chemoreceptors present in the lung and lower respiratory airways to the central cardiorespiratory center in the brain [126]. It is thus noteworthy that neuropeptides, such as CGRP and their G-protein coupled receptors (GPCRs), are present and play a prominent role not only in the lung but also in the brain area involved in ventilatory and cardiovascular regulation [135,136,137,138]. 

Although few studies have evaluated the integrative physiological actions of CGRP, it might be of interest to evaluate whether CGRP receptor-targeted gepants might provide not only symptomatic alleviation of COVID-19-related neurological symptoms but also beneficial central actions on ventilatory and cardiovascular disturbances accompanying COVID-19. Moreover, the recent realization that acute lung injury induces the release of CGRP, which in turn is implicated in COVID-19 symptoms, such as cough, fever, pain, and the release of IL-6, might illuminate a new scenario in which “gepants” might be used to target the SARS-CoV-2 nsp16/nsp10 complex while treating excessive lung inflammation caused by SARS-CoV-2. Because the neuronal system is responsible for pain transmission, inflammation, and immunomodulation throughout the entire pulmonary system, including the localized release of COVID-19-related cytokines [139], anti-inflammatory drugs previously employed for reducing inflammatory and neuroinflammatory pain, such as the fatty acid amide hydrolase inhibitor PF-04457845 [140,141,142] and the cyclooxygenase inhibitor antrafenine [143], might be repurposed to dually target the SARS-CoV-2 nsp16/nsp10 complex while reducing the pulmonary innervation system in ARDS associated with advanced COVID-19 disease. 

#### 4.3.2. TG-100801 and Lifitegrast

The best predicted candidate in terms of binding energy and stability targeting the unique nsp16/nsp10 interaction interface was TG-100801, the first topically applied, multitargeted VEGFR/Src kinase inhibitor to advance into the clinic. TG-100801 is administered noninvasively as an eye drop and has been designed to suppress VEGF-mediated leakage and additional kinase targets associated with inflammation, edema, and angiogenesis in several pathological eye conditions, including viral infections [144,145]. Lifitegrast, an FDA-approved drug for the treatment of dry eye syndrome [146,147], was also among the final list of strong nsp16/nsp10-targeting candidates. While the potential extra-respiratory transmissible routes of SARS-CoV-2 remain unclear, clinical entities, such as conjunctivitis, anterior uveitis, retinitis, and optic neuritis, have been documented in feline and murine coronavirus models. Indeed, SARS-CoV-2 might be transmitted via aerosol contact with the conjunctiva, causing conjunctival congestion as the initial symptom of COVID-19, and viral particles are present in ocular secretions [148,149,150]. Thus, because exposed mucous membranes and unprotected eyes appear to significantly increase the risk of SARS-CoV-2 transmission, the possibility of ocular SARS-CoV-2 infection cannot be ignored and the increase in awareness of eye protection is necessary. Indeed, dry eye, blurred vision, and foreign body sensation have been shown to rank as the top three COVID-19-related ocular symptoms and diseases [151]. The computational prediction of TG-100801 and lifitegrast as anti-SARS-CoV-2 drugs might have not only pharmacophoric implications for designing more specific compounds targeting the nsp16/nsp10 interface but could also provide a pharmacological approach of blocking eye-related transmission routes or treating SARS-CoV-2-driven ocular manifestations in patients. 

### 4.4. Decontamination and Self-Disinfection

#### Phthalocyanine

Phthalocyanine is an archetypal member of so-called photodynamic antimicrobials, in which the combination of a sensitizing drug and visible light causes selective destruction of viruses, bacteria, and other pathogens when applied to consumer or medical products [152,153,154,155]. Although the length of time that SARS-CoV-2 can survive on inanimate surfaces [156,157] varies depending on numerous factors, the risk of transmission of COVID-19 in social contact environments and non-healthcare work settings can be largely minimized through good general hygiene, including handwashing and wearing a face mask. A different scenario occurs in the so-called “infectious clean” following the discharge or transfer of patients with COVID-19 in healthcare facilities, which requires both thorough cleaning and disinfection for environmental decontamination. Currently, such procedures involve physical cleaning with detergent followed by disinfection with a hospital-grade disinfectant with activity against some viruses, or a chlorine-based product, such as sodium hypochlorite. Photochemotherapy using metal-derivatives of phthalocyanines, which might become activated even under weak indoor light, has been investigated for the disinfection of lipid envelope viruses, including HIV, hepatitis B virus, herpes simplex virus 1, and influenza A virus [H1N1] [152,153,154,155]. Our current prediction of phthalocyanine as a putative pan-inhibitor of the SAM-binding site, the nsp16/nsp10 interface, and the RNA-binding groove of the SARS-CoV-2 2′-O-MTase, might provide an additional biological layer of interest to the usage of phthalocyanine derivatives as biocidal molecules that can be incorporated into self-disinfecting materials, such as fabrics, films, and coatings, which can play a vital role in preventing the transmission of SARS-CoV-2, especially in healthcare settings.

We acknowledge that other candidates predicted to target the nsp16/nps10 2′-O-MTase might provide additional clinico-molecular advantages against SARS-CoV-2/COVID-19. Lifirafenib, an orally available dual RAF kinase/EGFR inhibitor with an acceptable risk–benefit profile [158], and more particularly, entrectinib (Rozlytrek, RDX-1; Genentech Inc.: South San Francisco, CA, USA), a potent oral inhibitor of the tyrosine kinase TRK A/B/C, ROS1, and ALK with a good safety profile [159,160,161,162], were predicted to highly stably interact with the nsp16/nsp10 interface and the (nsp10-stabilized) SAM-binding pocket, respectively. At a moment in which the oncology community is carefully balancing treatment decisions for lung cancer patients in light of the COVID-19 pandemic (e.g., withholding immunotherapy from patients whose tumors do not have known driver mutations; interactions of COVID-19 treatments in clinical trials, such as remdesivir and hydroxychloroquine, with immunotherapeutics and tyrosine kinase inhibitors), if COVID-19-positive oncologic patients would be treated with tyrosine kinase inhibitors, such as entrectinib, associating clinical outcomes with treatment with these drugs may merit investigation.

## 5. Conclusions

The COVID-19-causing virus SARS-CoV-2 has evolved distinct 2′-O’-MTase machinery to autonomously modify the 5′ cap of its RNA. Such 2′-O-methylation is a crucial mechanism used to subvert innate host antiviral responses by escaping interferon-stimulated proteins that are capable of detecting non-2′-O-methylated viral RNA and inhibiting its translation [40]. Although the previous biochemical and structural delineation of distinctive activating traits of nsp16 by nsp10 immediately suggested a new way to design and develop highly SARS-CoV-2-specific antivirals [45,46], very few attempts have been made to reactivate the innate immune restriction and antagonism of SARS-CoV-2 RNAs by pharmacologically preventing their RNA cap 2′-O-methylation. Here, we aimed to systematically identify repurposable drugs that we predict can target the RNA cap 2′-O-MTase nsp16/nsp10 protein complex but also other molecular and clinical traits specific to SARS-CoV-2 and COVID-19. Although we acknowledge that our final computational prediction might appear too wide for pre-clinical and clinical validation, we expect that by providing a multi-layer network framework (Figure 6), our findings should help to select the narrowest list of candidates that can be rapidly tested before evaluating their in vivo efficiency and side-effects. Indeed, some of the predicted drugs (e.g., tegobuvir, sonidegib, siramesine, antrafenine, bemcentinib, itacitinib, and phthalocyanine) might be suitable for rapid repurposing to pharmacologically reactivate the innate immune restriction and antagonism of SARS-CoV-2 RNAs lacking 2′-O-methylation.

## Supplementary Materials

The following are available online at https://www.mdpi.com/1999-4915/12/5/525/s1. Table S1: Binding energies and dissociation constants of top-scoring nsp16/nsp10-targeting candidates. Table S2: Drug candidates targeting the SAM-binding site of the nsp16/nsp10 protein complex. Table S3: Drug candidates targeting the nsp16/nsp10 interface of the nsp16/nsp10 protein complex. Table S4: Drug candidates targeting the RNA-binding groove of the nsp16/nsp10 protein complex. Table S5: Drug candidates targeting the SAM-binding site and nsp16/nsp10 interface of the nsp16/nsp10 protein complex. Table S6: Drug candidates targeting the SAM-binding site and RNA-binding groove of the nsp16/nsp10 protein complex. Table S7: Drug candidates targeting the nsp16/nsp10 interface and RNA-binding groove of the nsp16/nsp10 protein complex. Table S8: Drug candidates targeting the SAM-binding site, nsp16/nsp10 interface, and RNA-binding groove of the nsp16/nsp10 protein complex. Table S9: Filtering of nsp16/nsp10-targeting candidate drugs based on MD simulations for drug-protein complexes. Table S10: Selection of nsp16/nsp10-targeting candidate drugs based on MD simulations for drug-protein complexes.
