# Supplementary material for: Potential Drugs Targeting Early Innate Immune Evasion of SARS-Coronavirus 2 via 2’-O-Methylation of Viral RNA
Source: Viruses. 2020 May 10;12(5):525. doi: 10.3390/v12050525 (PMC7291090; doi:10.3390/v12050525)
Supplement: Supplementary file 1 [file viruses-12-00525-s001.pdf]

Supplementary Materials: Potential Drugs Targeting the SARS-Coronavirus 2 RNA Cap 2′-O-Methyltransferase nsp16/nsp10 Complex

José Antonio Encinar and Javier A. Menendez

Table S1. Binding energies and dissociation constants of top-scoring nsp16/nsp10-targeting candidates.

DB Code	Generic Name	SAM-Binding Site ΔG (kcal/mol)/K_D (μM)	Nsp16/nsp10 Interface ΔG (kcal/mol)/K_D (μM)	RNA-Binding Groove ΔG (kcal/mol)/K_D (μM)
DB00320	Dihydroergotamine	−10.8/0.0243		
DB03231	–	−10.3/0.0547		
DB06925	–	−10.3/0.0547		
DB08237	–	−10.3/0.0547		
DB11977	Golvatinib	−10.3/0.0547		
DB11986	Entrectinib	−10.3/0.0547		
DB12895	TD-139	−10.3/0.0547		
DB12899	TT-301	−10.3/0.0547		
DB13053	CP-195543	−10.3/0.0547		
DB14870	PF-5190457	−10.3/0.0547		
DB01897	–	−10.2/0.0643		
DB03571	–	−10.1/0.0757		
DB06638	Quarfloxin		−8.9/0.5309	
DB12799	Laniquidar		−8.9/0.5309	
DB06555	Siramesine		−8.5/1.0163	
DB05075	TG-100801		−8.4/1.1954	
DB13050	Tirilazad		−8.4/1.1954	
DB00872	Conivaptan		−8.4/1.1954	
DB14895	Vibegron		−8.4/1.1954	
DB06938	–		−8.3/1.4061	
DB11852	Tegobuvir		−8.2/1.654	
DB12341	Aticaprant		−8.2/1.654	
DB04289	Genz-10850		−8.2/1.654	
DB09143	Sonidegib		−8.1/1.9455	
DB04016	–			−9.8/0.1231
DB12457	Rimegepant			−9.7/0.1449
DB13109	PKI-179			−9.4/0.2358
DB01830	AP-22408			-9.4/0.2358
DB05678	SLx-4090			−9.3/0.2773

DB08233	–			−9.3/0.2773
DB08827	Lomitapide			−9.2/0.3262
DB03067	–			−9.2/0.3262
DB06896	-			−9.2/0.3262
DB15308	Ridinilazole			−9.1/0.3837
DB12154	Itacitinib			−9.1/0.3837
DB13042	Fenoverine			−9.0/0.4513
DB02449	-			−9.0/0.4513
DB15057	NUC-1031			−9.0/0.4513
DB12411	Bemcentinib	−10.9/0.0206	−8.5/1.0163	
DB05984	RAF-265	−10.2/0.0643	−8.3/1.4061	
DB01419	Antrafenine	−10.1/0.0757	−8.3/1.4061	
DB12012	PF-04457845	−10.4/0.0465		−9.1/0.3837
DB15382	SAR-125844		−8.2/1.654	−9.7/0.1449
DB12983	Phthalocyanine	−10.9/0.0206	−9.1/0.3837	−10.2/0.0643
DB12424	MK-3207	−10.9/0.0206	−8.5/1.0163	−9.7/0.1449
DB14773	Lifirafenib	−10.2/0.0643	−8.4/1.1954	−9.6/0.1704
DB11611	Lifitegrast	−10.3/0.0547	−8.3/1.4061	−9.0/0.4513

Table S2. Drug candidates targeting the SAM-binding site of the nsp16/nsp10 protein complex.

Compound Name	Compound Structure	Human Target	Drug Status
**Dihydroergotamine**	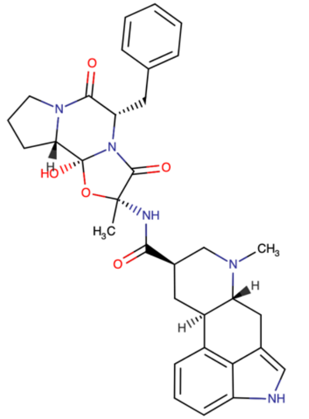	5-HT_{1D}a<br>5-HT_{1D}b receptors	Approved<br>(migraine)
**DB03231**<br>**(EXPT02670)**	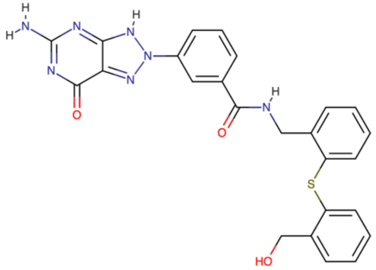	Dihydroneopterin aldolase<br>(*Staphylococcus aureus*)	Experimental

**DB06925**	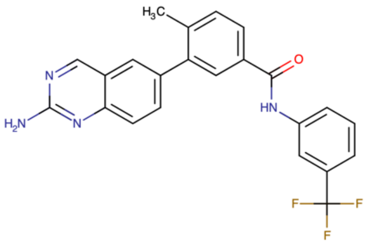	Tyrosine-protein kinase Lck	Experimental
**DB08237**	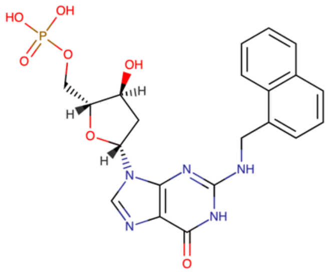	DNA polymerase kappa	Experimental
**Golvatinib**	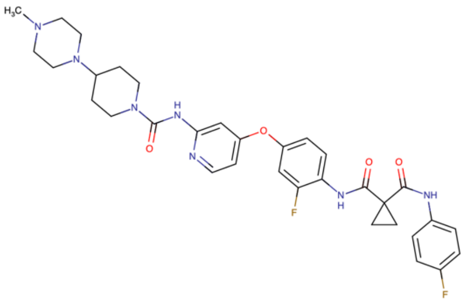	c-MET/VEGFR2	Investigational

**Entrectinib**	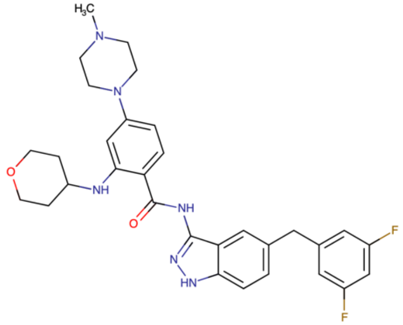	Tropomyosin receptor tyrosine kinases (TRKA, TRKB, TRKC)<br><br>Proto-oncogene tyrosine-protein kinase (ROS1)<br><br>Anaplastic lymphoma kinase (ALK)	Approved (ROS1-positive metastatic non-small cell lung cancer and NTRK gene fusion positive solid tumors)
**TD-139**	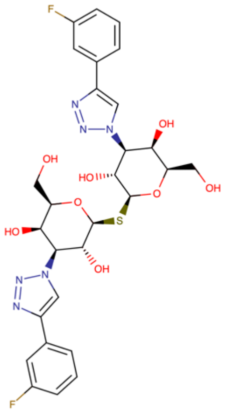	Galectin-3	Investigational
**TT-301**	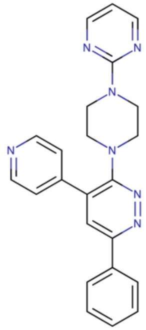	-	Investigational

**CP-195543**	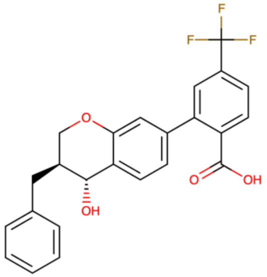	Leukotriene B4 receptor	Investigational
**PF-5190457**	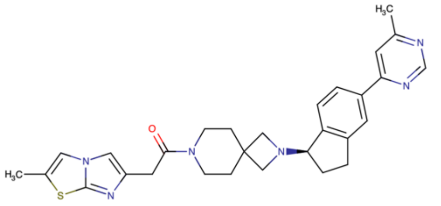	GH secretagogue receptor (GHS-R1a)	Investigational
**DB01897**	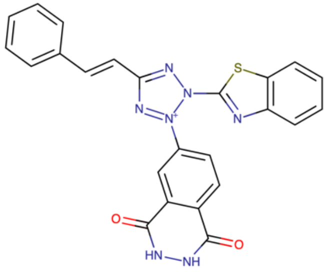	Hematopoietic prostaglandin D synthase	Experimental

**DB03571 (EXPT00198)**	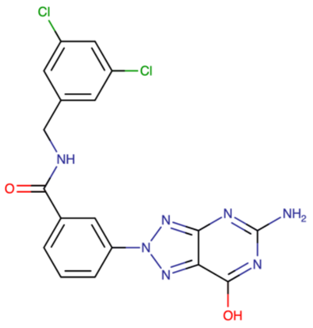	Dihydroneopterin aldolase (*Staphylococcus aureus*)	Experimental

Five of the predicted drugs were experimental ones, namely: DB03231 [3-(5-Amino-7-oxo-3,7-dihydro-2H-[1,2,3]triazolo[4,5-d]pyrimidin-2-yl)-*N*-(2-{[2-(hydroxymethyl)phenyl]sulfanyl}benzyl)benzamide)], DB06925 [3-(2-aminoquinazolin-6-yl-)-4-methyl-*N*-[3-(trifluoromethyl)phenyl]benzamine], and DB08237 [2′-deoxy-*N*-(naphtalen-1-ylmethyl)guanosine 5′-(dihydrogen phosphate)], DB01897 [2-(2f-benzothiazolyl)-5-styryl-3-(4f-phthalhydrazidyl)tetrazolium chloride], and DB03571 [3-(5-amino-7-hydroxy-[1,2,3]triazolo[4,5-d]pyrimidin-2-yl)-*N*-(3,5-dichlorobenzyl)-benzamide]. DB03231 is an *N*-benzylbenzamide targeting the dehydroneopterin aldolase activity in *Staphylococcus aureus*. DB06925 is a benzalnilide targeting the Lck protein, an Src family tyrosine kinase playing a critical role in T cell maturation and activation. DB08237 is a purine 2′-deoxyribonucleoside monophosphate targeting the DNA polymerase kappa specifically involved in DNA repair. DB01897 is a phthalazinone targeting the hematopoietic prostaglandin D synthase, a bifunctional enzyme catalyzing both the conversion of PGH2 to PGD2 and the conjugation of glutathione with aryl halides and organic isothiocyanates. DB03571 is an n-benzylbenzamide dehydroneopterin targeting aldolase activity in *S. aureus*. Five of the predicted drugs were investigational ones, namely: DB11977 (golvatinib), DB12895 (TD-139), DB12899 (TT-301), DB13053 (CP-195543), and DB14870 (PF-5190457). Golvatinib (also known as E7050) is a diarylether that potently inhibits c-Met and VEGFR-2 tyrosine kinases that has been proposed as a lead compound for anti-hepatitis A drug development [1–3]. TD-139 is a disaccharide that has been investigated for the treatment of idiopathic pulmonary fibrosis [4–6]. TT-301 is a phenylpyridazine that has been used in trials studying the treatment of traumatic brain injury [7]. CP-195543 is a linear diarylhepatanoid that potent and selectively inhibits leukotriene B4 (LTB4) receptor and has been used in trials studying the treatment of arthritis rheumatoid [8,9]. PF-5190457 is an orally bioavailable, potent, and selective GHS-R1a inverse agonist trialed to treat alcohol use disorder [10–12]. Two of the predicted compounds were FDA-approved drugs, namely: DB00320 (dihydroergotamine) and DB11986 (entrectinib). Dihydroergotamine is a 9,10 alpha-dihydro derivative of ergotamine that binds with high affinity to 5-HT$_{1D}$a and 5-HT1$_{1D}$b receptors and has been used for the therapy of migraine disorders by non-oral routes including an approved nasal spray formulation [13–15]. Entrectinib is an FDA-approved phenylpiparezine that functions as a tropomyosin receptor tyrosine kinase (TRK) TRKA, TRKB, TRKC, proto-oncogene tyrosine-protein kinase ROS1, and anaplastic lymphoma kinase (ALK) inhibitor in the treatment of ROS1-positive metastatic non-small-cell lung cancer and NTRK gene fusion-positive solid tumors [16–18].

Table S3. Drug candidates targeting the nsp16/nsp10 interface of the nsp16/nsp10 protein complex.

Compound Name	Compound Structure	Human Target	Drug Status
**Quarfloxin**	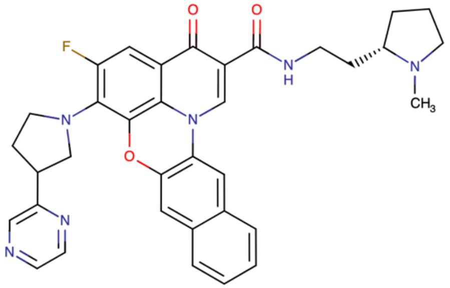	ribosomal RNA (rRNA) biogenesis	Investigational
**Laniquidar**	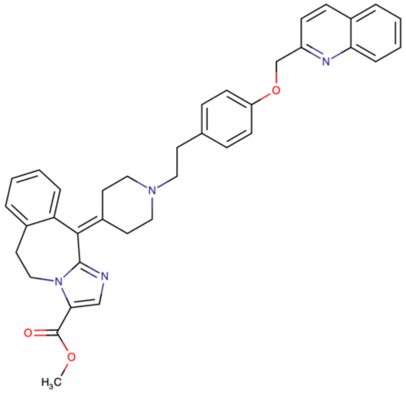	Multidrug resistance protein 1	Investigational

**Siramesine**	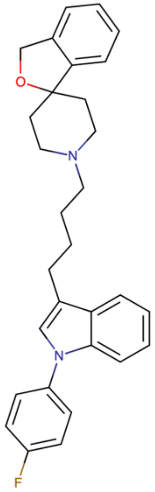	Sigma-2 receptor	Investigational
**TG-100801**	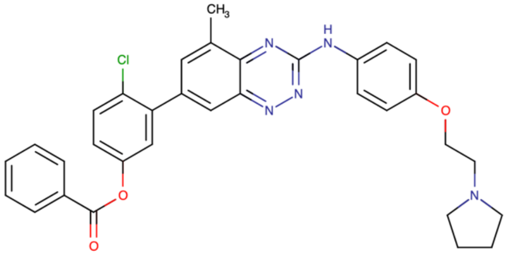	VEGFR1/2/3<br>Tyrosine-protein kinase CSK	Investigational

**Tirilazad**	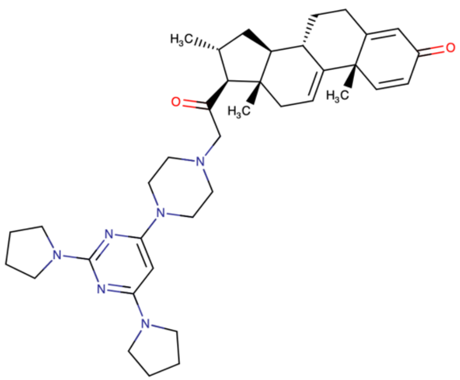	Lipid peroxidation	Investigational
**Conivaptan**	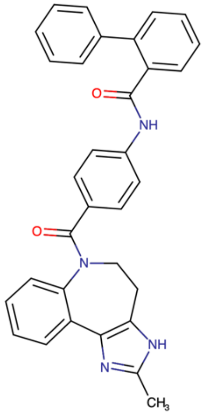	Vasopressin receptor (V1a and V2)	Approved (Hyponatremia, syndrome of inappropriate antidiuretic hormone)

**Vibegron**	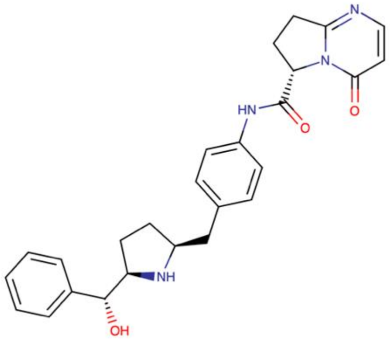	*Beta 3* adrenergic receptor (β3AR)	Investigational
**DB06938**	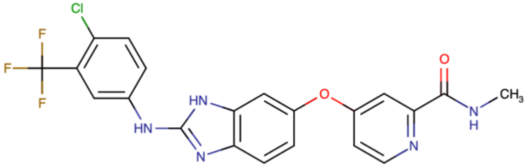	VEGFR2	Experimental
**Tegobuvir**	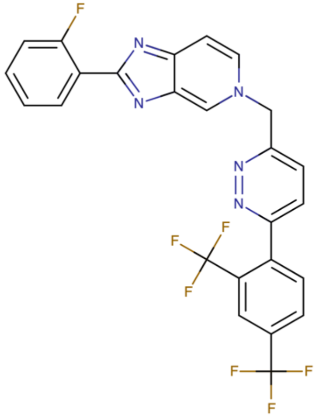	Hepatitis C Virus NS5B Polymerase	Investigational

**Aticaprant**	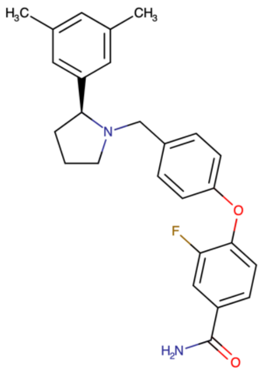	κ-opioid receptor (KOR)	Investigational
**Genz-10850**	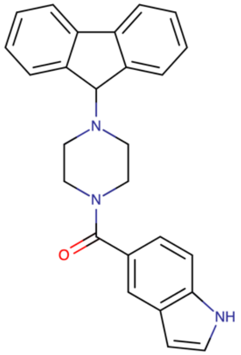	Enoyl-[acyl-carrier-protein] reductase (nadh) activity (*M. tuberculosis*)	Experimental
**Sonidegib**	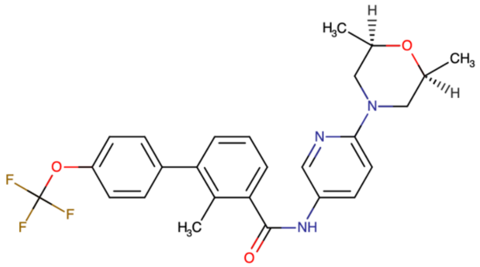	Smoothened (SMO)	Approved (Basal cell carcinoma)

Two of the predicted compounds were experimental drugs, namely: DB06938 [4-[[2-[[4-chloro-3-(trifluoromethyl)phenyl]amino]-3H-benzimidazol-5-yl]oxy]-*N*-methyl-pyridine-2-carboxamide], and DB04289 (Genz-10850). DB06938 is a diarylether targeting vascular endothelial growth factor receptor 2 (VEGFR2). Genz-10850 is a fluorene targeting the enoyl-[acyl-carrier-protein] reductase (nadh) activity of the mycobacterial type II fatty acid synthase (FAS-II) system [19,20]. Eight of the predicted compounds were investigational drugs, namely: DB06638 (quarfloxin, CX-3543), DB12799 (laniquidar), DB06555 (siramesine), DB05075 (TG-100801), DB13050 (tirilazad), DB14895 (vibegron), DB11852 (tegobuvir), and DB12341 (LY-2456302/Aticaprant). Quarfloxin is a phenoxazine that binds and stabilizes DNA G-quadruplex (G4) sequences and operates as a direct inhibitor of ribosomal RNA biogenesis [21–25]. Laniquidar is a benzazepine belonging to the 3rd generation of highly specific and potent P-glycoprotein inhibitors [26–28]. Siramesine is a phenylpyrrole that operates as a selective sigma-2 receptor agonist to induce lysosomal leakage, cytoprotective autophagosome accumulation, and ferroptosis [29–33]. TG-100801 is a depsidone that multitargets vascular endothelial growth factor receptor (VEGFR)/Src kinases-induced viral immunopathology [34,35]. Tirilazad is a nonglucocorticoid, 21-aminosteroid (lazaroid) that potently inhibits oxygen free radical-induced, iron-catalyzed, lipid peroxidation in stroke and chronic obstructive pulmonary disease (COPD) [36,37]. Vibegron (RVT-901/MK-4618/KRP-114V) is a beta-3 adrenergic receptor beta 3 (β_3AR) agonist employed for the treatment of overactive bladder [38–40]. Tegobuvir is a non-nucleoside phenylpyridazine targeting the hepatitis C Virus RNA-dependent RNA NS5B polymerase [41–44]. LY-2456302/Aticaprant is a diphenylether targeting the κ-opioid receptor (KOR) used as a candidate to treat major depressive disorder [45,46]. Two of the predicted compounds were FDA-approved drugs, namely: DB00872 (conivaptan) and DB09143 (sonidegib). Conivaptan is a benzalnilide targeting the vasopressin receptors V1a and V2 that has been approved for hyponatremia (low blood sodium levels) caused by syndrome of inappropriate antidiuretic hormone (SIADH) [47,48]. Sonidegib (also named Odomzo) is a biphenyl derivative that antagonizes smoothened (SMO) to block the hedgehog (Hh) pathway and has been approved by the FDA to treat basal cell carcinomas [49–51].

Table S4. Drug candidates targeting the RNA-binding groove of the nsp16/nsp10 protein complex.

Compound Name	Compound Structure	Human Target	Drug Status
**DB04016**	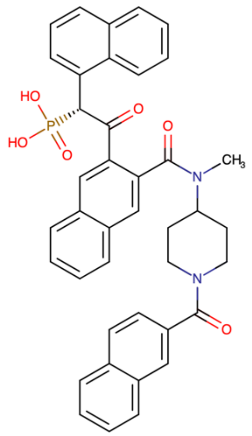	Cathepsin G	Experimental
**Rimegepant**	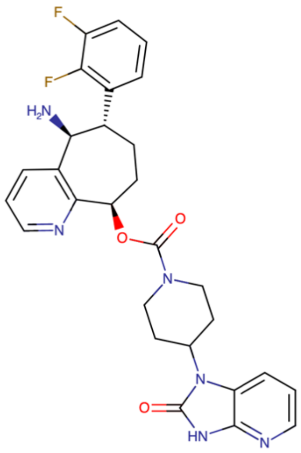	Calcitonin gene-related peptide type 1 receptor (CGRP receptor)	Approved (Migraine headache)

**PKI-179**	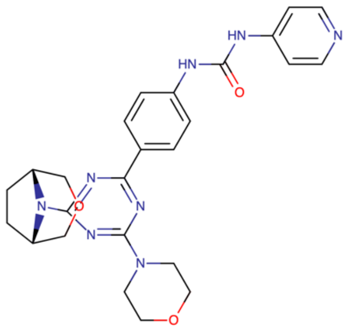	mTOR	Investigational
**AP-22408**	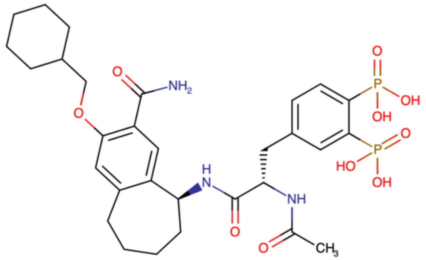	Tyrosine-protein kinase Lck	Experimental
**SLx-4090**	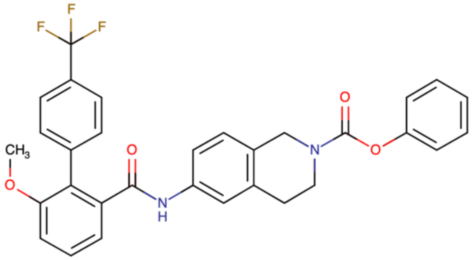	Microsomal triglyceride transfer protein large subunit	Investigational

**DB08233**	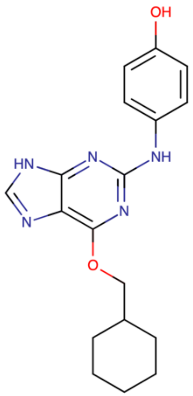	Cyclin-A2<br>Cyclin-dependent kinase 2	Experimental
**Lomitapide**	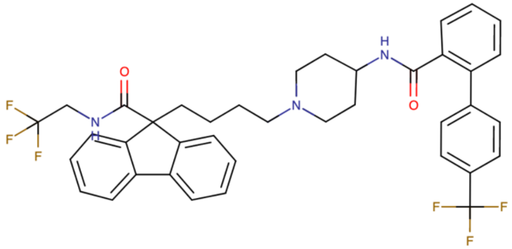	Microsomal triglyceride transfer protein large subunit	Approved<br>(homozygous familial hypercholesterolemia)

**DB03067**	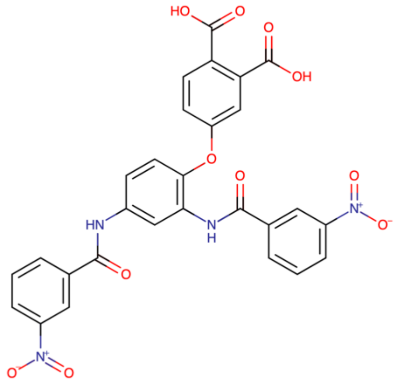	Glycogen phosphorylase, muscle form	Experimental
**DB06896**	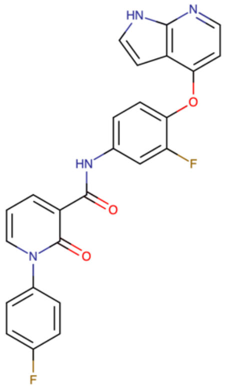	c-MET	Experimental

**Ridinilazole**	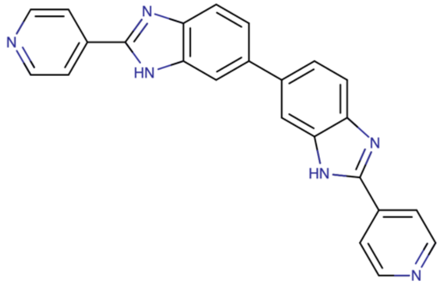	-	Investigational
**Itacitinib**	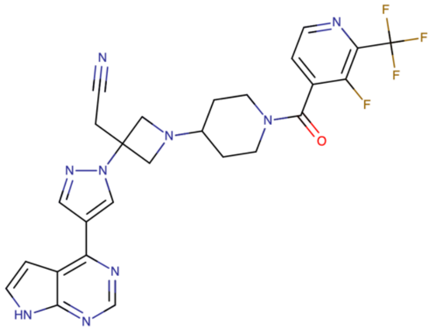	JAK1	Investigational

**Fenoverine**	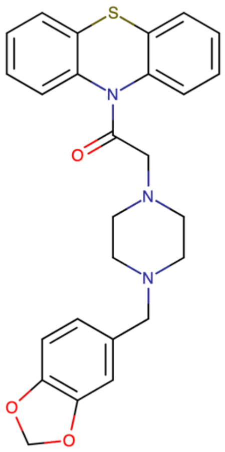	Calcium ions (Ca2+) channels	Investigational
**DB02449**	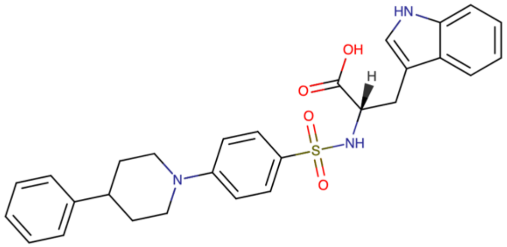	Stromelysin-1	Experimental

**Acelarin (NUC-1031)**	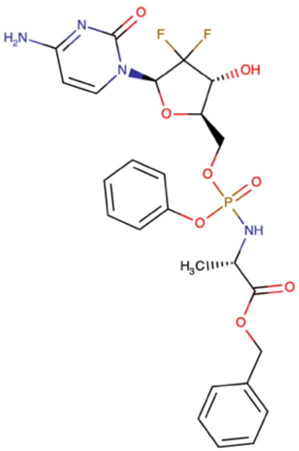	DNA	Investigational

Six of the predicted compounds were experimental drugs, namely: DB04016 [2-[3-({methyl[1-(2-naphthoyl)piperidin-4-yl]amino}carbonyl)-2-naphthyl]-1-(1-naphthyl)-2-oxoethylphosphonic acid], DB01830 (AP-22408), DB08233 [6-cyclohexylmethyloxy-2-(4′-hydroxyanilino)purine], DB03067 [4-{2,4-Bis[(3-Nitrobenzoyl)Amino]Phenoxy}Phthalic Acid], DB06896 [1-(4-fluorophenyl)-*N*-[3-fluoro-4-(1H-pyrrolo[2,3-b]pyridin-4-yloxy)phenyl]-2-oxo-1,2-dihydropyridine-3-carboxamide], DB02449 [3-(1h-indol-3-yl)-2-[4-(4-phenyl-piperidin-1-yl)-benzenesulfonylamino]-propionic acid]. DB04016 is a stilbene targeting the serine-type endopeptidase activity of cathepsin G. AP-22408 is a phenylalanine derivative targeting the Sh2 domain of the tyrosine-protein kinase Lck [52,53]. DB08233 is a hypoxanthine targeting cyclin-A2 and cyclin-dependent kinase 2. DB03067 is a benzanilide targeting pyridoxal phosphate binding of the glycogen phosphorylase. DB06896 is an aromatic anilide targeting the c-MET/hepatocyte growth factor receptor. DB02449 is a phenylpiperidine targeting stromelysin-1 (MMP3). Six of the predicted compounds were investigational drugs, namely: DB13109 (PKI-179), DB05678 (SLx-4090), DB15308 (ridinilazole), DB12154 (itacitinib), DB13042 (fenoverine), and DB15057 (acelarin/NUC-1031). PKI-179 is a *N*-phenylurea that dually targets the phosphatidylinositol-3-kinase (PI3K)/mammalian target of rapamycin (mTOR) pathway [54,55]. SLx-4090 is a microsomal triglyceride transfer protein (MTTP) inhibitor potentially for the treatment of type 2 diabetes [56]. Ridinilazole is a targeted-spectrum antimicrobial that shows potential in treatment of *Clostridium difficile* infection [57,58]. Itacitinib is a pyridinecarboxylic acid derivative that functions as an oral, selective inhibitor of the Janus Kinase (JAK) family of protein tyrosine kinases (TYKs) with selectivity for JAK1 in the treatment of inflammatory and neoplastic diseases [59,60]. Fenoverine is an old antispasmodic phenothiazine that inhibits calcium channel currents in smooth muscle cells [61]. Acelarin/NUC-1031, also known as fosgemcitabine palabenamide, is a pre-activated nucleotide analog (gemcitabine monophosphate) that incorporates a protective phosphoramidate moiety [62,63]. Two of the predicted compounds were FDA-approved drugs, namely: DB12457 (rimegepant) and DB08827 (lomitapide). Rimegepant is an imidazopyridine that functions as an oral antagonist of the CGRP receptor and has been approved for the acute treatment of migraine headache [64,65]. Lomitapide is a fluorene that directly inhibits microsomal triglyceride transfer protein (MTP) that has been approved as an orphan drug to reduce LDL cholesterol, total cholesterol, apolipoprotein B, and non-high-density lipoprotein (non-HDL) cholesterol in patients with homozygous familial hypercholesterolemia (HoFH) [66,67].

Table S5. Drug candidates targeting the SAM-binding site and nsp16/nsp10 interface of the nsp16/nsp10 protein complex.

Compound Name	Compound Structure	Human Target	Drug Status
**Bemcentinib**	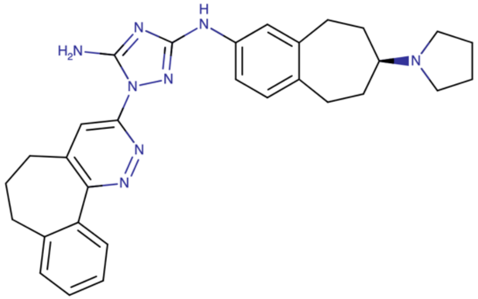	AXL receptor tyrosine kinase	Investigational
**RAF-265**	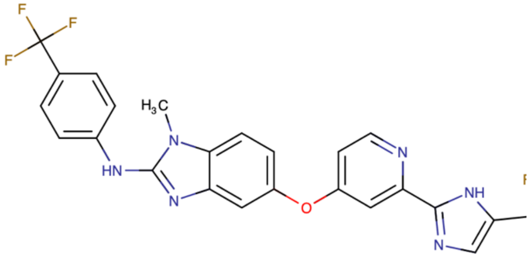	VEGFR2/BRAF	Investigational

**Antrafenine**	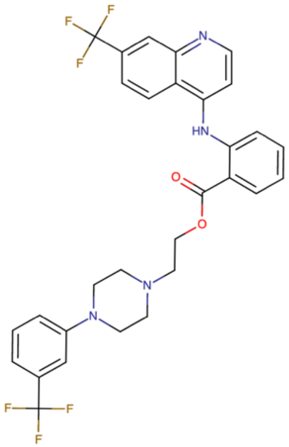	Prostaglandin G/H synthase	Approved (analgesic/anti-inflammatory)

R-428 (BGB324, bemcentinib) is an aralkylamine that selectively and potentially inhibits AXL, a receptor tyrosine kinase implicated in epithelial-to-mesenchymal transition (EMT), inflammation, fibrosis, and is a key suppressor of innate immune response [68–72]. RAF-265 is a potent RAF/VEGFR2 inhibitor [73]. Antrafenine is a piperazine derivative drug that acts as an analgesic and anti-inflammatory drug with similar efficacy to naproxen via inhibition of cyclooxygenase activity [74].

Table S6. Drug candidates targeting the SAM-binding site and RNA-binding groove of the nsp16/nsp10 protein complex.

Compound Name	Compound Structure	Human Target	Drug Status
**PF-04457845**	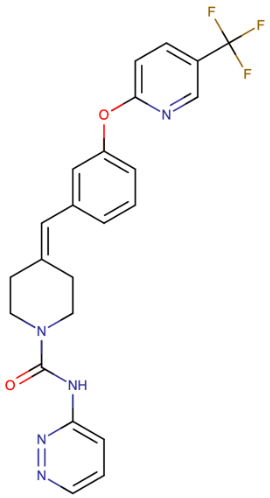	Fatty acid amide hydrolase (FAAH)	Investigational

PF-04457845 is a diarylether that targets the fatty acid amide hydrolase (FAAH) that has been investigated for the treatment of Tourette Syndrome and cannabis dependence and is under investigation in fear conditioning [75–77].

Table S7. Drug candidates targeting the nsp16/nsp10 interface and RNA-binding groove of the nsp16/nsp10 protein complex.

Compound Name	Compound Structure	Human Target	Drug Status
**SAR-125844**	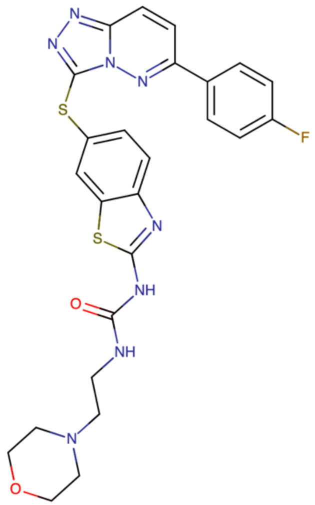	MET receptor tyrosine kinase	Investigational

SAR-125844 is a potent intravenously active and highly selective MET tyrosine kinase inhibitor with potential antineoplastic activity [78].

Table S8. Drug candidates targeting the SAM-binding site, nsp16/nsp10 interface, and RNA-binding groove of the nsp16/nsp10 protein complex.

Compound Name	Compound Structure	Human Target	Drug Status
**Phthalocyanine**	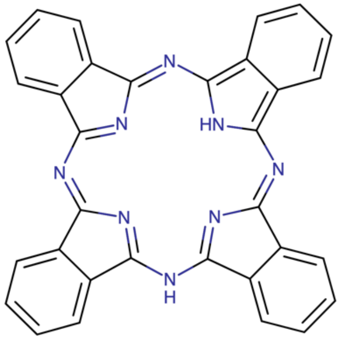	-	Investigational
**MK-3207**	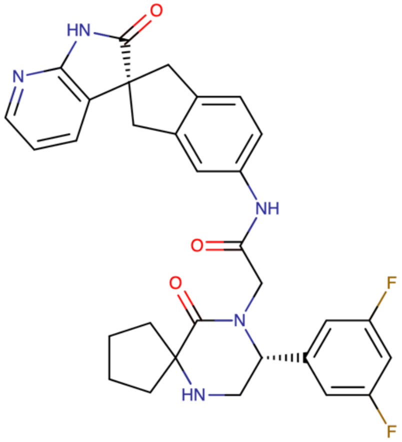	Calcitonin gene-related peptide type 1 receptor (CGRP receptor)	Investigational

**Lifirafenib (BGB-283)**	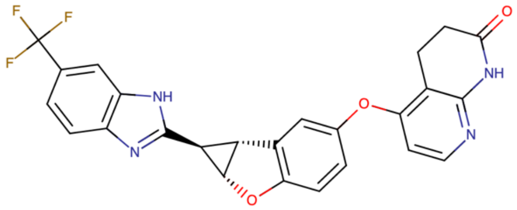	RAF/EGFR	Investigational
**Lifitegrast**	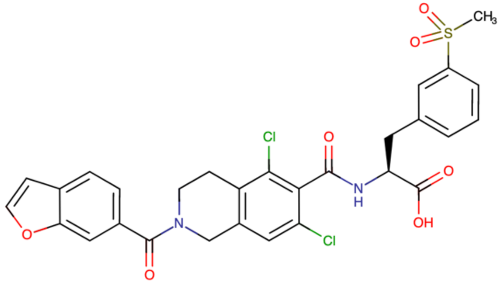	Integrin alpha-L	Approved (keratoconjunctivitis sicca)

Phthalocyanine is a cyclic tetrapyrrole that contains a phthalocyanine skeleton, which consists of four isoindole-type units, with the connecting carbon atoms in the macrocycle replaced by nitrogen. Metal derivatives of phthalocyanine have been studied for their photodynamic biocidal capacity against enveloped viruses [79–82]. MK-3207 is a potent and orally active CGRP receptor antagonists that has been used in trials studying the treatment of migraine and migraine disorders [83–85]. Lifirafenib (BGB-283) selectively binds to and inhibits the activity of wild-type BRAF and certain BRAF mutant forms, and EGFR [86]. Lifitegrast is a tetrahydroisoquinoline derivative that binds to the integrin lymphocyte function-associated antigen-1 (LFA-1), a cell surface protein found on leukocytes and blocks the interaction of LFA-1 with its cognate ligand intercellular adhesion molecule-1 (ICAM-1). Lifitegrast is a FDA approved drug for the treatment of keratoconjunctivitis sicca (dry eye syndrome) [87–89].

Table S9. Filtering of nsp16/nsp10-targeting candidate drugs based on MD simulations for drug-protein complexes.

Drug Name	Score	Target Site	MM∣PBSA (0–100 ns)	MM∣PBSA (last 30 ns)
DB06638/Quarfloxin	Weak	interface	9.102	9.568
DB06555/Siramesine	Strong	interface	39.69	48.014
DB05075/TG-100801	Strong	interface	61.668	69.891
DB13050/Tirilazad	Strong	interface	48.234	40.536
DB14895/Vibegron	Diminished	interface	−1.081	−5.227
DB05984/RAF-265	Diminished	interface	29.497	24.329
DB01419/Antrafenine	Strong	interface	40.95	44.568
DB12411/R-248/BGB-324	Strong	interface	24.997	27.923
DB12411/R-248/BGB-324	Diminished	SAM site	−3.615	4.448
DB11611/Lifitegrast	Diminished	interface	20.631	4.487
DB11611/Lifitegrast	Strong	SAM site	17.475	35.27
DB14773/Lifirafenib/BGB-283	Strong	interface	41.142	45.654
DB14773/Lifirafenib/BGB-283	Weak	RNA groove	7.821	13.649
DB00872/Conivaptan	Strong	interface	37.337	36.79
DB00872/Conivaptan	Diminished	RNA groove	7.314	17.604
DB12983/Phthalocyanine	Strong	interface	26.945	27.097
DB12983/Phthalocyanine	Weak	SAM site	9.904	14.088
DB12983/Phthalocyanine	Weak	RNA groove	0.823	7.984
DB12424/MK-3207	Strong	interface	30.874	20.984
DB12424/MK-3207	Weak	SAM site	17.778	26.56
DB12424/MK-3207	Diminished	RNA groove	5.05	13.097
DB00320/Dihydroergotamine	Weak	SAM site	7.543	12.095
DB12012/PF-04457845	Diminished	SAM site	24.106	31.865
DB03231	Weak	SAM site	1.923	11.759
DB06925	Weak	SAM site	6.514	10.686
DB08237	Diminished	SAM site	−42.994	−38.728
DB11977/Golvatinib	Diminished	SAM site	−5.329	6.381
DB11986/Entrectinib	Strong	SAM site	72.21	125.784
DB12895/TD-139	Diminished	SAM site	−16.553	−10.838
DB12899/TT-301	Diminished	SAM site	2.758	8.633
DB13053/CP-195543	Diminished	SAM site	18.46	20.945

DB14870/PF-5190457	Diminished	SAM site	28.169	80.869
DB14883/Lorecivivint	Diminished	RNA groove	46.902	21.97
DB13109/PKI-179	Diminished	RNA groove	12.456	17.272
DB11913/LY-2090314	Diminished	RNA groove	5.886	13.979
DB04016	Diminished	RNA groove	−24.172	−25.95
DB04739	Diminished	RNA groove	8.834	6.933
DB12154/Itacitinib	Strong	RNA groove	13.693	23.338
DB14859/Fosifloxouridine nafalbenamide	Diminished	RNA groove	−14.878	−17.657
DB06976	Strong	RNA groove	57.909	149.99
DB06844	Diminished	RNA groove	3,298	11,678
DB06938	Diminished	interface	18,613	10,329
DB11852/Tegobuvir	Strong	interface	55,989	56,05
DB12341/LY-2456302	Strong	interface	63.729	65.275
DB04289/Genz-10850	Strong	interface	27.67	28.451
DB09143/Sonidegib	Strong	interface	45.066	50.818
DB01897	Diminished	SAM site	24.505	28.252
DB03571	Strong	SAM site	16.184	23.292
DB12457/Rimegepant	Weak	RNA groove	5.958	18.991
DB01830/AP-22408	Diminished	RNA groove	−6.86	6.74
DB05678/SLx-4090	Diminished	RNA groove	17.443	9.449
DB08233	Diminished	RNA groove	12.558	19.145
DB08827/Lomitapide	Diminished	RNA groove	7.442	5.842
DB03067	Strong	RNA groove	72.626	72.934
DB15308/Ridinilazole	Strong	RNA groove	19.615	28.397
DB13042/Fenoverine	Diminished	RNA groove	6.763	16.032
DB02449 -9.0 kcal/mol	Strong	RNA groove	77.708	108.207
DB15057/Gemcitabine-phosphoramidate	Diminished	RNA groove	−15.739	3.958
DB05984/RAF-265	Diminished	SAM site	−3.582	−7.312
DB01419/Antrafenine	Strong	SAM site	83.067	162.432
DB15382/SAR-125844	Strong	interface	38.082	35.447
DB15382/SAR-125844	Weak	RNA groove	−0.584	12.192
DB12012/PF-04457845	Strong	RNA groove	30.717	68.413
DB14773/Lifirafenib/BGB-283	Weak	RNA groove	12.094	10.759
DB11611/Lifitegrast	Weak	RNA groove	12.339	19.104

DB11852/Tegobuvir	Strong	SAM site	34.551	44.181
DB11852/Tegobuvir	Diminished	RNA groove	25.524	30.613

Table S10. Selection of nsp16/nsp10-targeting candidate drugs based on MD simulations for drug-protein complexes.

Drug	Score	Target Site	MM I PBSA 0–100 ns (kcal/mol)	MM I PBSA last 30 ns (kcal/mol)
**DB01419/Antrafenine**	Strong	SAM-binding site	83.067	162.432
**DB11986/Entrectinib**	Strong	SAM-binding site	72.21	125.784
**DB02449**	Strong	RNA-binding groove	77.708	108.207
**DB03067**	Strong	RNA-binding groove	72.626	72.934
**DB12012/PF-04457845**	Strong	RNA-binding groove	30.717	68.413
**DB05075/TG-100801**	Strong	nsp10-nsp16 interface	61.668	69.891
**DB12341/Aticaprant**	Strong	nsp10-nsp16 interface	63.729	65.275
**DB11852/Tegobuvir**	Strong	nsp10-nsp16 interface	55.989	56.05
**DB09143/Sonidegib**	Strong	nsp10-nsp16 interface	45.066	50.818
**DB06555/Siramesine**	Strong	nsp10-nsp16 interface	39.69	48.014
**DB14773/Lifirafenib/BGB-283**	Strong	nsp10-nsp16 interface	41.142	45.654
**DB01419/Antrafenine**	Strong	nsp10-nsp16 interface	40.95	44.568
**DB13050/Tirilazad**	Strong	nsp10-nsp16 interface	48.234	40.536
**DB00872/Conivaptan**	Strong	nsp10-nsp16 interface	37.337	36.79
**DB15382/SAR-125844**	Strong	nsp10-nsp16 interface	38.082	35.447
**DB04289/Genz-10850**	Strong	nsp10-nsp16 interface	27.67	28.451
**DB12411/Bemcentinib**	Strong	nsp10-nsp16 interface	24.997	27.923
**DB12983/Phthalocyanine**	Strong	nsp10-nsp16 interface	26.945	27.097
**DB15308/Ridinilazole**	Strong	RNA-binding groove	19.615	28.397
**DB11611/Lifitegrast**	Strong	SAM-binding site	17.475	35.27
**DB12424/MK-3207**	Strong	nsp10-nsp16 interface	30.874	20.984
**DB12154/Itacitinib**	Strong	RNA-binding groove	13.693	23.338
DB06638/Quarfloxin	Weak	nsp10-nsp16 interface	9.102	9.568
DB11611/Lifitegrast	Weak	RNA-binding groove	12.339	19.104
DB12457/Rimegepant	Weak	RNA-binding groove	5.958	18.991
DB14773/Lifirafenib/BGB-283	Weak	RNA-binding groove	12.094	10.759
DB15382/SAR-125844	Weak	RNA-binding groove	-0.584	12.192
DB12983/Phthalocyanine	Weak	RNA-binding groove	0.823	7.984
DB12424/MK-3207	Weak	SAM-binding site	17.778	26.56

DB03571	Weak	SAM-binding site	16.184	23.292
DB12983/Phthalocyanine	Weak	SAM-binding site	9.904	14.088
DB00320/Dihydroergotamine	Weak	SAM-binding site	7.543	12.095
DB03231	Weak	SAM-binding site	1.923	11.759

References

1. 1. Nakagawa T, Tohyama O, Yamaguchi A, Matsushima T, Takahashi K, Funasaka S, Shirotori S, Asada M, Obaishi H. E7050: a dual c-Met and VEGFR-2 tyrosine kinase inhibitor promotes tumor regression and prolongs survival in mouse xenograft models. Cancer Sci. 2010;101:210-5.
2. 2. Molife LR, Dean EJ, Blanco-Codesido M, Krebs MG, Brunetto AT, Greystoke AP, Daniele G, Lee L, Kuznetsov G, Myint KT, Wood K, de Las Heras B, Ranson MR. A phase I, dose-escalation study of the multitargeted receptor tyrosine kinase inhibitor, golvatinib, in patients with advanced solid tumors. Clin Cancer Res. 2014;20:6284-94.
3. 3. Cao L, Liu P, Yang P, Gao Q, Li H, Sun Y, Zhu L, Lin J, Su D, Rao Z, Wang X. Structural basis for neutralization of hepatitis A virus informs a rational design of highly potent inhibitors. PLoS Biol. 2019;17:e3000229.
4. 4. Mackinnon AC, Gibbons MA, Farnworth SL, Leffler H, Nilsson UJ, Delaine T, Simpson AJ, Forbes SJ, Hirani N, Gauldie J, Sethi T. Regulation of transforming growth factor-β1-driven lung fibrosis by galectin-3. Am J Respir Crit Care Med. 2012;185:537-46.
5. 5. Volarevic V, Milovanovic M, Ljujic B, Pejnovic N, Arsenijevic N, Nilsson U, Leffler H, Lukic ML. Galectin-3 deficiency prevents concanavalin A-induced hepatitis in mice. Hepatology. 2012;55:1954-64.
6. 6. Saksida T, Nikolic I, Vujicic M, Nilsson UJ, Leffler H, Lukic ML, Stojanovic I, Stosic-Grujicic S. Galectin-3 deficiency protects pancreatic islet cells from cytokine-triggered apoptosis in vitro. J Cell Physiol. 2013;228:1568-76.
7. 7. James ML, Wang H, Cantillana V, Lei B, Kernagis DN, Dawson HN, Klaman LD, Laskowitz DT. TT-301 inhibits microglial activation and improves outcome after central nervous system injury in adult mice. Anesthesiology. 2012;116:1299-311.
8. 8. Showell HJ, Conklyn MJ, Alpert R, Hingorani GP, Wright KF, Smith MA, Stam E, Salter ED, Scampoli DN, Meltzer S, Reiter LA, Koch K, Piscopio AD, Cortina SR, Lopez-Anaya A, Pettipher ER, Milici AJ, Griffiths RJ. The preclinical pharmacological profile of the potent and selective leukotriene B4 antagonist CP-195543. J Pharmacol Exp Ther. 1998;285:946-54.
9. 9. Caron S. The synthesis of CP-195543, an LTB4 antagonist for the treatment of inflammatory diseases. Curr Opin Drug Discov Devel. 1999;2:550-6
10. 10. Bhattacharya SK, Andrews K, Beveridge R, Cameron KO, Chen C, Dunn M, Fernando D, Gao H, Hepworth D, Jackson VM, Khot V, Kong J, Kosa RE, Lapham K, Loria PM, Londregan AT, McClure KF, Orr ST, Patel J, Rose C, Saenz J, Stock IA, Storer G, VanVolkenburg M, Vrieze D, Wang G, Xiao J, Zhang Y. Discovery of PF-5190457, a Potent, Selective, and Orally Bioavailable Ghrelin Receptor Inverse Agonist Clinical Candidate. ACS Med Chem Lett. 2014;5:474-9.
11. 11. Lee MR, Tapocik JD, Ghareeb M, Schwandt ML, Dias AA, Le AN, Cobbina E, Farinelli LA, Bouhlal S, Farokhnia M, Heilig M, Akhlaghi F, Leggio L. The novel ghrelin receptor inverse agonist PF-5190457 administered with alcohol: preclinical safety experiments and a phase 1b human laboratory study. Mol Psychiatry. 2020;25:461-475.
12. 12. Lee MR, Farokhnia M, Cobbina E, Saravanakumar A, Li X, Battista JT, Farinelli LA, Akhlaghi F, Leggio L. Endocrine effects of the novel ghrelin receptor inverse agonist PF-5190457: Results from a placebo-controlled human laboratory alcohol co-administration study in heavy drinkers. Neuropharmacology. 2019 Sep 23:107788.
13. 13. Silberstein SD, Kori SH. Dihydroergotamine: a review of formulation approaches for the acute treatment of migraine. CNS Drugs. 2013;27:385-94.
14. 14. Silberstein SD, Shrewsbury SB, Hoekman J. Dihydroergotamine (DHE) - Then and Now: A Narrative Review. Headache. 2020;60:40-57.
15. 15. Albrecht D, Iwashima M, Dillon D, Harris S, Levy J. A Phase 1, Randomized, Open-Label, Safety, Tolerability, and Comparative Bioavailability Study of Intranasal Dihydroergotamine Powder (STS101), Intramuscular Dihydroergotamine Mesylate, and Intranasal DHE Mesylate Spray in Healthy Adult Subjects. Headache. 2020;60:701-712.
16. 16. Drilon A, Siena S, Ou SI, Patel M, Ahn MJ, Lee J, Bauer TM, Farago AF, Wheler JJ, Liu SV, Doebele R, Giannetta L, Cerea G, Marrapese G, Schirru M, Amatu A, Bencardino K, Palmeri L, Sartore-Bianchi A, Vanzulli A, Cresta S, Damian S, Duca M, Ardini E, Li G, Christiansen J, Kowalski K, Johnson AD, Patel R, Luo D, Chow-Maneval E, Hornby Z, Multani PS, Shaw AT, De Braud FG. Safety and Antitumor Activity

of the Multitargeted Pan-TRK, ROS1, and ALK Inhibitor Entrectinib: Combined Results from Two Phase I Trials (ALKA-372-001 and STARTRK-1). Cancer Discov. 2017;7:400-409.

17. 17. Drilon A, Siena S, Dziadziuszko R, Barlesi F, Krebs MG, Shaw AT, de Braud F, Rolfo C, Ahn MJ, Wolf J, Seto T, Cho BC, Patel MR, Chiu CH, John T, Goto K, Karapetis CS, Arkenau HT, Kim SW, Ohe Y, Li YC, Chae YK, Chung CH, Otterson GA, Murakami H, Lin CC, Tan DSW, Prenen H, Riehl T, Chow-Maneval E, Simmons B, Cui N, Johnson A, Eng S, Wilson TR, Doebele RC; trial investigators. Entrectinib in ROS1 fusion-positive non-small-cell lung cancer: integrated analysis of three phase 1-2 trials. Lancet Oncol. 2020;21:261-270.
18. 18. Doebele RC, Drilon A, Paz-Ares L, Siena S, Shaw AT, Farago AF, Blakely CM, Seto T, Cho BC, Tosi D, Besse B, Chawla SP, Bazhenova L, Krauss JC, Chae YK, Barve M, Garrido-Laguna I, Liu SV, Conkling P, John T, Fakih M, Sigal D, Loong HH, Buchschacher GL Jr, Garrido P, Nieva J, Steuer C, Overbeck TR, Bowles DW, Fox E, Riehl T, Chow-Maneval E, Simmons B, Cui N, Johnson A, Eng S, Wilson TR, Demetri GD; trial investigators. Entrectinib in patients with advanced or metastatic NTRK fusion-positive solid tumours: integrated analysis of three phase 1-2 trials. Lancet Oncol. 2020;21:271-282.
19. 19. Kuo MR, Morbidoni HR, Alland D, Sneddon SF, Gourlie BB, Staveski MM, Leonard M, Gregory JS, Janjigian AD, Yee C, Musser JM, Kreiswirth B, Iwamoto H, Perozzo R, Jacobs WR Jr, Sacchettini JC, Fidock DA. Targeting tuberculosis and malaria through inhibition of Enoyl reductase: compound activity and structural data. J Biol Chem. 2003;278:20851-9.
20. 20. Chollet A, Mori G, Menendez C, Rodriguez F, Fabing I, Pasca MR, Madacki J, Korduláková J, Constant P, Quémard A, Bernardes-Génisson V, Lherbet C, Baltas M. Design, synthesis and evaluation of new GEQ derivatives as inhibitors of InhA enzyme and Mycobacterium tuberculosis growth. Eur J Med Chem. 2015;101:218-35.
21. 21. Drygin D, Siddiqui-Jain A, O'Brien S, Schwaebe M, Lin A, Bliesath J, Ho CB, Proffitt C, Trent K, Whitten JP, Lim JK, Von Hoff D, Anderes K, Rice WG. Anticancer activity of CX-3543: a direct inhibitor of rRNA biogenesis. Cancer Res. 2009;69:7653-61.
22. 22. Xu H, Di Antonio M, McKinney S, Mathew V, Ho B, O'Neil NJ, Santos ND, Silvester J, Wei V, Garcia J, Kabeer F, Lai D, Soriano P, Banáth J, Chiu DS, Yap D, Le DD, Ye FB, Zhang A, Thu K, Soong J, Lin SC, Tsai AH, Osako T, Algara T, Saunders DN, Wong J, Xian J, Bally MB, Brenton JD, Brown GW, Shah SP, Cescon D, Mak TW, Caldas C, Stirling PC, Hieter P, Balasubramanian S, Aparicio S. CX-5461 is a DNA G-quadruplex stabilizer with selective lethality in BRCA1/2 deficient tumours. Nat Commun. 2017;8:14432.
23. 23. Balasubramanian S, Hurley LH, Neidle S. Targeting G-quadruplexes in gene promoters: a novel anticancer strategy? Nat Rev Drug Discov. 2011;10:261-75.
24. 24. Kerry LE, Pegg EE, Cameron DP, Budzak J, Poortinga G, Hannan KM, Hannan RD, Rudenko G. Selective inhibition of RNA polymerase I transcription as a potential approach to treat African trypanosomiasis. PLoS Negl Trop Dis. 2017;11:e0005432.
25. 25. Harris LM, Monsell KR, Noulin F, Famodimu MT, Smargiasso N, Damblon C, Horrocks P, Merrick CJ. G-Quadruplex DNA Motifs in the Malaria Parasite Plasmodium falciparum and Their Potential as Novel Antimalarial Drug Targets. Antimicrob Agents Chemother. 2018;62:pii: e01828-17.
26. 26. Thomas H, Coley HM. Overcoming multidrug resistance in cancer: an update on the clinical strategy of inhibiting p-glycoprotein. Cancer Control. 2003;10:159-65.
27. 27. Werle M, Takeuchi H, Bernkop-Schnürch A. New-generation efflux pump inhibitors. Expert Rev Clin Pharmacol. 2008;1:429-40.
28. 28. Joshi P, Vishwakarma RA, Bharate SB. Natural alkaloids as P-gp inhibitors for multidrug resistance reversal in cancer. Eur J Med Chem. 2017;138:273-292.
29. 29. Ostenfeld MS, Fehrenbacher N, Høyer-Hansen M, Thomsen C, Farkas T, Jäättelä M. Effective tumor cell death by sigma-2 receptor ligand siramesine involves lysosomal leakage and oxidative stress. Cancer Res. 2005;65:8975-83.
30. 30. Ostenfeld MS, Høyer-Hansen M, Bastholm L, Fehrenbacher N, Olsen OD, Groth-Pedersen L, Puustinen P, Kirkegaard-Sørensen T, Nylandsted J, Farkas T, Jäättelä M. Anti-cancer agent siramesine is a lysosomotropic detergent that induces cytoprotective autophagosome accumulation. Autophagy. 2008;4:487-99.
31. 31. Zhitomirsky B, Yunaev A, Kreiserman R, Kaplan A, Stark M, Assaraf YG. Lysosomotropic drugs activate TFEB via lysosomal membrane fluidization and consequent inhibition of mTORC1 activity. Cell Death Dis. 2018;9:1191.

32. 32. Ma S, Henson ES, Chen Y, Gibson SB. Ferroptosis is induced following siramesine and lapatinib treatment of breast cancer cells. Cell Death Dis. 2016;7:e2307.
33. 33. Villalpando-Rodriguez GE, Blankstein AR, Konzelman C, Gibson SB. Lysosomal Destabilizing Drug Siramesine and the Dual Tyrosine Kinase Inhibitor Lapatinib Induce a Synergistic Ferroptosis through Reduced Heme Oxygenase-1 (HO-1) Levels. Oxid Med Cell Longev. 2019;2019:9561281.
34. 34. Palanki MS, Akiyama H, Campochiaro P, Cao J, Chow CP, Dellamary L, Doukas J, Fine R, Gritzen C, Hood JD, Hu S, Kachi S, Kang X, Klebansky B, Kousba A, Lohse D, Mak CC, Martin M, McPherson A, Pathak VP, Renick J, Soll R, Umeda N, Yee S, Yokoi K, Zeng B, Zhu H, Noronha G. Development of prodrug 4-chloro-3-(5-methyl-3-{[4-(2-pyrrolidin-1-ylethoxy)phenyl]amino}-1,2,4-benzotriazin-7-yl)phenyl benzoate (TG100801): a topically administered therapeutic candidate in clinical trials for the treatment of age-related macular degeneration. J Med Chem. 2008;51:1546-59.
35. 35. Sharma S, Mulik S, Kumar N, Suryawanshi A, Rouse BT. An anti-inflammatory role of VEGFR2/Src kinase inhibitor in herpes simplex virus 1-induced immunopathology. J Virol. 2011;85:5995-6007.
36. 36. Sena E, Wheble P, Sandercock P, Macleod M. Systematic review and meta-analysis of the efficacy of tirilazad in experimental stroke. Stroke. 2007;38:388-94.
37. 37. Flessas II, Papalois AE, Toutouzas K, Zagouri F, Zografos GC. Effects of lazaroids on intestinal ischemia and reperfusion injury in experimental models. J Surg Res. 2011;166:265-74.
38. 38. Edmondson SD, Zhu C, Kar NF, Di Salvo J, Nagabukuro H, Sacre-Salem B, Dingley K, Berger R, Goble SD, Morriello G, Harper B, Moyes CR, Shen DM, Wang L, Ball R, Fitzmaurice A, Frenkl T, Gichuru LN, Ha S, Hurley AL, Jochnowitz N, Levorse D, Mistry S, Miller RR, Ormes J, Salituro GM, Sanfiz A, Stevenson AS, Villa K, Zamlynny B, Green S, Struthers M, Weber AE. Discovery of Vibegron: A Potent and Selective β3 Adrenergic Receptor Agonist for the Treatment of Overactive Bladder. J Med Chem. 2016;59:609-23.
39. 39. Yoshida M, Takeda M, Gotoh M, Yokoyama O, Kakizaki H, Takahashi S, Masumori N, Nagai S, Minemura K. Efficacy of vibegron, a novel β3-adrenoreceptor agonist, on severe urgency urinary incontinence related to overactive bladder: post hoc analysis of a randomized, placebo-controlled, double-blind, comparative phase 3 study. BJU Int. 2020 Jan 28. doi: 10.1111/bju.15020. [Epub ahead of print]
40. 40. Staskin D, Frankel J, Varano S, Shortino D, Jankowich R, Mudd PN Jr. International Phase III, Randomized, Double-Blind, Placebo- and Active-Controlled Study to Evaluate the Safety and Efficacy of Vibegron in Patients with Symptoms of Overactive Bladder: EMPOWUR. J Urol. 2020 Mar 11:101097JU0000000000000807.

41. Hebner CM, Han B, Brendza KM, Nash M, Sulfab M, Tian Y, Hung M, Fung W, Vivian RW, Trenkle J, Taylor J, Bjornson K, Bondy S, Liu X, Link J, Neyts J, Sakowicz R, Zhong W, Tang H, Schmitz U. The HCV non-nucleoside inhibitor Tegobuvir utilizes a novel mechanism of action to inhibit NS5B polymerase function. PLoS One. 2012;7:e39163.

41. 42. Wyles DL, Rodriguez-Torres M, Lawitz E, Shiffman ML, Pol S, Herring RW, Massetto B, Kanwar B, Trenkle JD, Pang PS, Zhu Y, Mo H, Brainard DM, Subramanian GM, McHutchison JG, Habersetzer F, Sulkowski MS. All-oral combination of ledipasvir, vedroprevir, tegobuvir, and ribavirin in treatment-naïve patients with genotype 1 HCV infection. Hepatology. 2014;60:56-64.
42. 43. Vliegen I, Paeshuyse J, Zhong W, Neyts J. In vitro combinations containing Tegobuvir are highly efficient in curing cells from HCV replicon and in delaying/preventing the development of drug resistance. Antiviral Res. 2015;120:112-21.
43. 44. Liu M, Xu Q, Guo S, Zuo R, Hong Y, Luo Y, Li Y, Gong P, Liu Y. Design, synthesis, and structure-activity relationships of novel imidazo[4 ,5-c]pyridine derivatives as potent non-nucleoside inhibitors of hepatitis C virus NS5B. Bioorg Med Chem. 2018;26:2621-2631.
44. 45. Li W, Sun H, Chen H, Yang X, Xiao L, Liu R, Shao L, Qiu Z. Major Depressive Disorder and Kappa Opioid Receptor Antagonists. Transl Perioper Pain Med. 2016;1:4-16.
45. 46. Page S, Mavrikaki MM, Lintz T, Puttick D, Roberts E, Rosen H, Carroll FI, Carlezon WA, Chartoff EH. Behavioral Pharmacology of Novel Kappa Opioid Receptor Antagonists in Rats. Int J Neuropsychopharmacol. 2019;22:735-745. 100. Ali F, Raufi MA, Washington B, Ghali JK. Conivaptan: a dual vasopressin receptor v1a/v2 antagonist [corrected]. Cardiovasc Drug Rev. 2007;25:261-79.
46. 47. Ali F, Raufi MA, Washington B, Ghali JK. Conivaptan: a dual vasopressin receptor v1a/v2 antagonist [corrected]. Cardiovasc Drug Rev. 2007;25:261-79.

47. 48. Ghali JK, Farah JO, Daifallah S, Zabalawi HA, Zmily HD. Conivaptan and its role in the treatment of hyponatremia. Drug Des Devel Ther. 2009;3:253-68.
48. 49. Burness CB. Sonidegib: First Global Approval. Drugs. 2015;75:1559-66.
49. 50. Collier NJ, Ali FR, Lear JT. The safety and efficacy of sonidegib for the treatment of locally advanced basal cell carcinoma. Expert Rev Anticancer Ther. 2016;16:1011-8.
50. 51. Jain S, Song R, Xie J. Sonidegib: mechanism of action, pharmacology, and clinical utility for advanced basal cell carcinomas. Onco Targets Ther. 2017;10:1645-1653.
51. 52. Shakespeare W, Yang M, Bohacek R, Cerasoli F, Stebbins K, Sundaramoorthi R, Azimioara M, Vu C, Pradeepan S, Metcalf C 3rd, Haraldson C, Merry T, Dalgarno D, Narula S, Hatada M, Lu X, van Schravendijk MR, Adams S, Violette S, Smith J, Guan W, Bartlett C, Herson J, Iuliucci J, Weigele M, Sawyer T. Structure-based design of an osteoclast-selective, nonpeptide src homology 2 inhibitor with in vivo antiresorptive activity. Proc Natl Acad Sci U S A. 2000;97:9373-8.
52. 53. Rucci N, Susa M, Teti A. Inhibition of protein kinase c-Src as a therapeutic approach for cancer and bone metastases. Anticancer Agents Med Chem. 2008;8:342-9.
53. 54. Venkatesan AM, Chen Z, dos Santos O, Dehnhardt C, Santos ED, Ayral-Kaloustian S, Mallon R, Hollander I, Feldberg L, Lucas J, Yu K, Chaudhary I, Mansour TS. PKI-179: an orally efficacious dual phosphatidylinositol-3-kinase (PI3K)/mammalian target of rapamycin (mTOR) inhibitor. Bioorg Med Chem Lett. 2010;20:5869-73.
54. 55. Mohd Rehan. A structural insight into the inhibitory mechanism of an orally active PI3K/mTOR dual inhibitor, PKI-179 using computational approaches. J Mol Graph Model. 2015;62:226-234.
55. 56. Kim E, Campbell S, Schueller O, Wong E, Cole B, Kuo J, Ellis J, Ferkany J, Sweetnam P. A small-molecule inhibitor of enterocytic microsomal triglyceride transfer protein, SLx-4090: biochemical, pharmacodynamic, pharmacokinetic, and safety profile. J Pharmacol Exp Ther. 2011;337:775-85.
56.
57. Carlson TJ, Endres BT, Bassères E, Gonzales-Luna AJ, Garey KW. Ridinilazole for the treatment of Clostridioides difficile infection. Expert Opin Investig Drugs. 2019;28:303-310.
57. 58. Cho JC, Crotty MP, Pardo J. Ridinilazole: a novel antimicrobial for *Clostridium difficile* infection. Ann Gastroenterol. 2019;32:134-140.
58. 59. Roskoski R Jr. Janus kinase (JAK) inhibitors in the treatment of inflammatory and neoplastic diseases. Pharmacol Res. 2016;111:784-803.
59. 60. Gong X, Darpo B, Xue H, Punwani N, He K, Barbour AM, Epstein N, Landman R, Chen X, Yeleswaram S. Evaluation of Clinical Cardiac Safety of Itacitinib, a JAK1 Inhibitor, in Healthy Participants. Clin Pharmacol Drug Dev. 2019 Dec 10. doi: 10.1002/cpdd.758. [Epub ahead of print]
60. 61. Gonella J, Lalanne C, Mironneau J. Fenoverine: a novel synchronizer of smooth muscle motility by interference with cellular calcium flow. Curr Med Res Opin. 1987;10:427-35.
61. 62. Slusarczyk M, Lopez MH, Balzarini J, Mason M, Jiang WG, Blagden S, Thompson E, Ghazaly E, McGuigan C. Application of ProTide technology to gemcitabine: a successful approach to overcome the key cancer resistance mechanisms leads to a new agent (NUC-1031) in clinical development. J Med Chem. 2014;57:1531-42.
62. 63. Blagden SP, Rizzuto I, Suppiah P, O'Shea D, Patel M, Spiers L, Sukumaran A, Bharwani N, Rockall A, Gabra H, El-Bahrawy M, Wasan H, Leonard R, Habib N, Ghazaly E. Anti-tumour activity of a first-in-class agent NUC-1031 in patients with advanced cancer: results of a phase I study. Br J Cancer. 2018;119:815-822.
63. 64. Lipton RB, Croop R, Stock EG, Stock DA, Morris BA, Frost M, Dubowchik GM, Conway CM, Coric V, Goadsby PJ. Rimegepant, an Oral Calcitonin Gene-Related Peptide Receptor Antagonist, for Migraine. N Engl J Med. 2019;381:142-149.
64. 65. Croop R, Goadsby PJ, Stock DA, Conway CM, Forshaw M, Stock EG, Coric V, Lipton RB. Efficacy, safety, and tolerability of rimegepant orally disintegrating tablet for the acute treatment of migraine: a randomised, phase 3, double-blind, placebo-controlled trial. Lancet. 2019;394:737-745.
65. 66. Cuchel M, Bloedon LT, Szapary PO, Kolansky DM, Wolfe ML, Sarkis A, Millar JS, Ikewaki K, Siegelman ES, Gregg RE, Rader DJ. Inhibition of microsomal triglyceride transfer protein in familial hypercholesterolemia. N Engl J Med. 2007;356:148-56.
66. 67. Cuchel M, Meagher EA, du Toit Theron H, Blom DJ, Marais AD, Hegele RA, Averna MR, Sirtori CR, Shah PK, Gaudet D, Stefanutti C, Vigna GB, Du Plessis AM, Propert KJ, Sasiela WJ, Bloedon LT, Rader DJ; Phase 3 HoFH Lomitapide Study investigators. Efficacy and safety of a microsomal triglyceride transfer

protein inhibitor in patients with homozygous familial hypercholesterolaemia: a single-arm, open-label, phase 3 study. Lancet. 2013;381:40-6.

67. 68. Holland SJ, Pan A, Franci C, Hu Y, Chang B, Li W, Duan M, Torneros A, Yu J, Heckrodt TJ, Zhang J, Ding P, Apatira A, Chua J, Brandt R, Pine P, Goff D, Singh R, Payan DG, Hitoshi Y. R428, a selective small molecule inhibitor of Axl kinase, blocks tumor spread and prolongs survival in models of metastatic breast cancer. Cancer Res. 2010;70:1544-54.

68. 69. Sadahiro H, Kang KD, Gibson JT, Minata M, Yu H, Shi J, Chhipa R, Chen Z, Lu S, Simoni Y, Furuta T, Sabit H, Zhang S, Bastola S, Yamaguchi S, Alsheikh H, Komarova S, Wang J, Kim SH, Hambardzumyan D, Lu X, Newell EW, DasGupta B, Nakada M, Lee LJ, Nabors B, Norian LA, Nakano I. Activation of the Receptor Tyrosine Kinase AXL Regulates the Immune Microenvironment in Glioblastoma. Cancer Res. 2018;78:3002-3013.

69. 70. Ludwig KF, Du W, Sorrelle NB, Wnuk-Lipinska K, Topalovski M, Toombs JE, Cruz VH, Yabuuchi S, Rajeshkumar NV, Maitra A, Lorens JB, Brekken RA. Small-Molecule Inhibition of Axl Targets Tumor Immune Suppression and Enhances Chemotherapy in Pancreatic Cancer. Cancer Res. 2018;78:246-255

70. 71. Landolt L, Furriol J, Babickova J, Ahmed L, Eikrem Ø, Skogstrand T, Scherer A, Suliman S, Leh S, Lorens JB, Gausdal G, Marti HP, Osman T. AXL targeting reduces fibrosis development in experimental unilateral ureteral obstruction. Physiol Rep. 2019;7:e14091.

71. 72. Tutusaus A, de Gregorio E, Cucarull B, Cristóbal H, Aresté C, Graupera I, Coll M, Colell A, Gausdal G, Lorens JB, García de Frutos P, Morales A, Marí M. A Functional Role of GAS6/TAM in Nonalcoholic Steatohepatitis Progression Implicates AXL as Therapeutic Target. Cell Mol Gastroenterol Hepatol. 2020;9:349-368.

72. 73. James J, Ruggeri B, Armstrong RC, Rowbottom MW, Jones-Bolin S, Gunawardane RN, Dobrzanski P, Gardner MF, Zhao H, Cramer MD, Hunter K, Nepomuceno RR, Cheng M, Gitnick D, Yazdanian M, Insko DE, Ator MA, Apuy JL, Faraoni R, Dorsey BD, Williams M, Bhagwat SS, Holladay MW. CEP-32496: a novel orally active BRAF(V600E) inhibitor with selective cellular and in vivo antitumor activity. Mol Cancer Ther. 2012;11:930-41.

73. 74. Berry H, Coquelin JP, Gordon A, Seymour D. Antrafenine, naproxen and placebo in osteoarthritis: a comparative study. Br J Rheumatol. 1983;22:89-94.

74. 75. Johnson DS, Stiff C, Lazerwith SE, Kesten SR, Fay LK, Morris M, Beidler D, Liimatta MB, Smith SE, Dudley DT, Sadagopan N, Bhattachar SN, Kesten SJ, Nomanbhoy TK, Cravatt BF, Ahn K. Discovery of PF-04457845: A Highly Potent, Orally Bioavailable, and Selective Urea FAAH Inhibitor. ACS Med Chem Lett. 2011;2:91-96.

75. 76. Ahn K, Smith SE, Liimatta MB, Beidler D, Sadagopan N, Dudley DT, Young T, Wren P, Zhang Y, Swaney S, Van Becelaere K, Blankman JL, Nomura DK, Bhattachar SN, Stiff C, Nomanbhoy TK, Weerapana E, Johnson DS, Cravatt BF. Mechanistic and pharmacological characterization of PF-04457845: a highly potent and selective fatty acid amide hydrolase inhibitor that reduces inflammatory and noninflammatory pain. J Pharmacol Exp Ther. 2011;338:114-24.

76. 77. D'Souza DC, Cortes-Briones J, Creatura G, Bluez G, Thurnauer H, Deaso E, Bielen K, Surti T, Radhakrishnan R, Gupta A, Gupta S, Cahill J, Sherif MA, Makriyannis A, Morgan PT, Ranganathan M, Skosnik PD. Efficacy and safety of a fatty acid amide hydrolase inhibitor (PF-04457845) in the treatment of cannabis withdrawal and dependence in men: a double-blind, placebo-controlled, parallel group, phase 2a single-site randomised controlled trial. Lancet Psychiatry. 2019;6:35-45.

77. 78. Angevin E, Spitaleri G, Rodon J, Dotti K, Isambert N, Salvagni S, Moreno V, Assadourian S, Gomez C, Harnois M, Hollebecque A, Azaro A, Hervieu A, Rihawi K, De Marinis F. A first-in-human phase I study of SAR125844, a selective MET tyrosine kinase inhibitor, in patients with advanced solid tumours with MET amplification. Eur J Cancer. 2017;87:131-139.

78. 79. Donnelly RF, McCarron PA, Tunney MM. Antifungal photodynamic therapy. Microbiol Res. 2008;163:1-12.

79. 80. Nikolaeva-Glomb L, Mukova L, Nikolova N, Kussovski V, Doumanova L, Mantareva V, Angelov I, Wöhrle D, Galabov AS. Photodynamic Effect of some Phthalocyanines on Enveloped and Naked Viruses. Acta Virol. 2017;61:341-346.

80. 81. Remichkova M, Mukova L, Nikolaeva-Glomb L, Nikolova N, Doumanova L, Mantareva V, Angelov I, Kussovski V, Galabov AS. Virus inactivation under the photodynamic effect of phthalocyanine zinc(II) complexes. Z Naturforsch C J Biosci. 2017;72:123-128.

81. 82. Korneev D, Kurskaya O, Sharshov K, Eastwood J, Strakhovskaya M. Ultrastructural Aspects of Photodynamic Inactivation of Highly Pathogenic Avian H5N8 Influenza Virus. Viruses. 2019;11:pii: E955.
82. 83. Bell IM, Gallicchio SN, Wood MR, Quigley AG, Stump CA, Zartman CB, Fay JF, Li CC, Lynch JJ, Moore EL, Mosser SD, Prueksaritanont T, Regan CP, Roller S, Salvatore CA, Kane SA, Vacca JP, Selnick HG. Discovery of MK-3207: A Highly Potent, Orally Bioavailable CGRP Receptor Antagonist. ACS Med Chem Lett. 2010;1:24-9.
83. 84. Hewitt DJ, Aurora SK, Dodick DW, Goadsby PJ, Ge YJ, Bachman R, Taraborelli D, Fan X, Assaid C, Lines C, Ho TW. Randomized controlled trial of the CGRP receptor antagonist MK-3207 in the acute treatment of migraine. Cephalalgia. 2011;31:712-22.
84. 85. Crowley BM, Stump CA, Nguyen DN, Potteiger CM, McWherter MA, Paone DV, Quigley AG, Bruno JG, Cui D, Culberson JC, Danziger A, Fandozzi C, Gauvreau D, Kemmerer AL, Menzel K, Moore EL, Mosser SD, Reddy V, White RB, Salvatore CA, Kane SA, Bell IM, Selnick HG, Fraley ME, Burgey CS. Novel oxazolidinone calcitonin gene-related peptide (CGRP) receptor antagonists for the acute treatment of migraine. Bioorg Med Chem Lett. 2015;25:4777-4781.
85. 86. Desai J, Gan H, Barrow C, Jameson M, Atkinson V, Haydon A, Millward M, Begbie S, Brown M, Markman B, Patterson W, Hill A, Horvath L, Nagrial A, Richardson G, Jackson C, Friedlander M, Parente P, Tran B, Wang L, Chen Y, Tang Z, Huang W, Wu J, Zeng D, Luo L, Solomon B. Phase I, Open-Label, Dose-Escalation/Dose-Expansion Study of Lifirafenib (BGB-283), an RAF Family Kinase Inhibitor, in Patients With Solid Tumors. J Clin Oncol. 2020 Mar 17:JCO1902654.
86. 87. Zhong M, Gadek TR, Bui M, Shen W, Burnier J, Barr KJ, Hanan EJ, Oslob JD, Yu CH, Zhu J, Arkin MR, Evanchik MJ, Flanagan WM, Hoch U, Hyde J, Prabhu S, Silverman JA, Wright J. Discovery and Development of Potent LFA-1/ICAM-1 Antagonist SAR 1118 as an Ophthalmic Solution for Treating Dry Eye. ACS Med Chem Lett. 2012;3:203-6.
87. 88. Abidi A, Shukla P, Ahmad A. Lifitegrast: A novel drug for treatment of dry eye disease. J Pharmacol Pharmacother. 2016;7:194-198.
88. 89. Paton DM. Lifitegrast: First LFA-1/ICAM-1 antagonist for treatment of dry eye disease. Drugs Today (Barc). 2016;52:485-493.
